# Emerging dynamics pathways of response and resistance to PD-1 and CTLA-4 blockade: tackling uncertainty by confronting complexity

**DOI:** 10.1186/s13046-021-01872-3

**Published:** 2021-02-18

**Authors:** Allan Relecom, Maysaloun Merhi, Varghese Inchakalody, Shahab Uddin, Darawan Rinchai, Davide Bedognetti, Said Dermime

**Affiliations:** 1 Department of Medical Oncology, Translational Research Institute, National Center for Cancer Care and Research, Hamad Medical Corporation, Doha, Qatar; 2 Translational Research Institute & Dermatology Institute, Academic Health System, Hamad Medical Corporation, Doha, Qatar; 3 Cancer Research Program, Research Branch, Sidra Medicine, Doha, Qatar; 4 Department of Internal Medicine and Medical Specialties, University of Genoa, Genoa, Italy; 5 College of Health and Life Sciences, Hamad Bin Khalifa University, Doha, Qatar

**Keywords:** Dynamics biomarkers, Immune checkpoint inhibitors, PD-1, CTLA-4, Tregs, MDSCs

## Abstract

Immune checkpoint inhibitors provide considerable therapeutic benefit in a range of solid cancers as well as in a subgroup of hematological malignancies. Response rates are however suboptimal, and despite considerable efforts, predicting response to immune checkpoint inhibitors ahead of their administration in a given patient remains elusive. The study of the dynamics of the immune system and of the tumor under immune checkpoint blockade brought insight into the mechanisms of action of these therapeutic agents. Equally relevant are the mechanisms of adaptive resistance to immune checkpoint inhibitors that have been uncovered through this approach. In this review, we discuss the dynamics of the immune system and of the tumor under immune checkpoint blockade emanating from recent studies on animal models and humans. We will focus on mechanisms of action and of resistance conveying information predictive of therapeutic response.

## Background

Immune checkpoint inhibitors (ICIs) are at the forefront of a therapeutic revolution in the treatment of cancer. Acting by modifying the anti-tumoral immune response, they offer the attractive prospect of long-lasting and self-sustained responses, having proven their potential to elicit considerable tumor control in a range of solid tumors as well as in a subgroup of hematological malignancies. Five year overall survival rates reaching 44 % in stage IV melanoma patients treated with an anti-PD-1 monotherapy in first line is in bold contrast with the 8 % 5 year overall survival rates observed under chemotherapy in the same pathology [1, 2], illustrating the considerable potential of these therapeutic agents. Reported response rates to ICIs are however limited, ranging from 10 to 40 % in monotherapy [3, 4]. Despite this large disparity in patient outcome, ICIs are, with very few exceptions, administered on a one treatment fits all basis. Moving towards a more potent and tailored approach to ICI prescription calls for a better understanding of the mechanisms underlying the action of these agents.

Several parameters influencing the probability of response to ICIs have been identified and recently reviewed elsewhere [5]. These can be subdivided into features of the tumor genome [6, 7], host immune-related traits [8] and the microbiota [9–11]. These multiple and diverse factors shape the complex interaction of the tumor and the immune system. Furthermore, tumor evolution rests on somatic mutations, clonal selection and random genetic drift [12, 13], making their precise course of development difficult to predict. Stochastic processes were also shown to determine the behavior of the immune system [14, 15]. As such, the tumor, the immune system and their common interaction, have to be considered as a complex dynamic processes, the behavior of which not being fully predictable based on their state at a given point in time [16].

In this review, we intend to highlight the importance of studying mechanisms influencing response to ICIs under the scope of the dynamics of the immune system and of the tumor under treatment. We will focus on the multitude of immune cell subsets that were shown to be impacted by ICIs. We will also discuss tumor-intrinsic mechanisms of immune evasion in an effort to provide a comprehensive review of identified dynamic mechanisms leveraging or infringing the response to these agents. Our discussion will emphasize on the two subclasses of ICIs registered for use in a range of solid tumor types, namely cytotoxic T-lymphocyte antigen 4 (CLTA-4) and programmed cell death protein 1/ programmed death-ligand 1 (PD-1/PD-L1) inhibitors, reporting on studies conducted on both animal models and humans. The large scope of research covered required a focus on the results of a selected set of the most relevant studies in the field.

## Effector T cells under anti-CTLA-4

Following its upregulation on T cells upon T cell receptor (TCR) engagement, CTLA-4 binds with its CD80 and CD86 ligands, thereby outcompeting the CD28 co-stimulatory receptor [17]. The CTLA-4 checkpoint is understood to mainly regulate T cell activation during the priming phase, B7 ligands being constitutively, but not exclusively, expressed by antigen presenting cells (APC). By infringing on this mechanism of T cell intrinsic negative regulation, CTLA-4 blockade leads to enhanced T cell proliferation and activation, as illustrated by the rapidly lethal lymphoproliferation displayed by CTLA-4 deficient mouse [18]. In tumor bearing mouse models, this action of CTLA-4 blockade translates into the expansion of both CD4 + and CD8 + effector T cells in the tumor microenvironment (TME)[19]. In these models, the increase in CD4 + T cells in the tumor appears to be of a greater magnitude than that of CD8 + T cells [19], both T cell subsets proving necessary in mediating tumor immune control [20]. CD8 + T cells expanding in the TME under anti-CTLA-4 express markers of exhaustion (PD-1, T cell immunoglobulin and mucin domain-containing protein 3 [Tim-3]), activation (glucocorticoid-induced tumor necrosis factor receptor [GITR], CD38) and co-stimulation/development (CD27 and CD127) molecules [20, 21]. These reinvigorated CD8 + T cells are, at least to some extent, tumor-antigen specific [20, 22, 23] and their expansion in the TME under anti-CTLA-4 correlates with response in a number of human studies [24, 25]. In line with these findings, Ji et al. reported that melanoma lesions responding to ipilimumab (anti-CTLA-4) displayed a greater on-treatment increase in transcripts consistent with an interferon gamma (IFN-γ) induced T cell cytotoxic response compared to non-responding ones [26]. Enhanced memory CD8 + T cell expansion can also be observed under CTLA-4 blockade [27]. This is expected to be critical for long-term tumor control and is reported to be predictive of treatment benefit in clinical studies [28, 29]. Of note, a study on advanced melanoma patients treated by the anti-CTLA-4 tremelimumab reports no association between the on-treatment expansion of CD8 + effector T cells observed in the TME under treatment and response [30]. Interestingly, CD8 + T cells of non-responding lesions showed similar activation profiles as those infiltrating responding ones [30]. Although the small sample size of the study, which had only 3 objective responses, paired with the more limited benefit observed under tremelimumab monotherapy as compared to ipilimumab in clinical studies, has to be taken into account, these findings suggest the importance of counteracting immunosuppressive forces in the TME [31, 32]. It also highlights the precept that adequate CD8 + effector T cell function is necessary but not sufficient for tumor growth suppression under immunotherapy [33].

The main CD4 + T cell subset expanding in tumors of mouse models under CTLA-4 blockade displays a Th1-like effector phenotype and distinctively expresses ICOS, which is a marker of T follicular helper cells [19]. Several observations account for the functional relevance of this T cell subset in the mediation anti-CTLA-4 action on the tumor. It has been shown that ICOS-deficient mice displayed attenuated anti-tumor T cell responses to anti-CTLA-4 therapy [34]. Also, a tumor microenvironment skewed in favor of a CD4 + T helper 1 (Th1) effector T cell infiltration is conditional for the response of castration resistant prostate cancer metastasis to anti-CTLA-4 [35]. Similarly, the expression of Th1 associated genes was reported to be higher in melanoma tumors of patients responding to ipilimumab compared to non-responders, and was documented to increase on-treatment [26]. Conversely, a peripheral blood profile skewed in favor of Th17 instead of Th1 cells was reported to be predictive of autoimmune toxicity rather than response to anti-CTLA-4 [36]. This is consistent with the documented pathogenic role played by Th17 polarization in a range of auto-immune diseases [37]. Inducible T cell co-stimulator (ICOS+) CD4 + T cells were shown to expand in the tumor and peripheral blood of anti-CTLA-4 treated patients in several clinical studies covering a range of solid tumor types [38–44], although their frequency in the periphery was not found to be impacted by CTLA-4 blockade in 2 others [28, 29]. Two clinical studies show the peripheral expansion of this T cell subset to be positively associated with response and/or overall survival following ipilimumab treatment [40, 41], although a third study reports no correlation of ICOS + CD4 + T cell dynamics with survival [29].

A proliferative surge can be observed in CD4 + and CD8 + T cells circulating in the peripheral blood of patients as early as 3 weeks after a first dose of anti-CTLA-4 [45–47]. The expansion of CD4 + and CD8 + T cells in the peripheral blood of patients under anti-CLTA-4 coincides with a shift towards a higher proportion of activated human leukocyte antigen (HLA)-DR^+^ relative to naïve C-C chemokine receptor type 7 (CCR7)^+^/CD45RA^+^) cells, as well as in an increased representation of central memory (CCR7^+^/CD45RA^−^) and effector memory (CCR7^−^/CD45RA^−^) cells [28, 42, 48–50]. Enhanced T cell responses against known tumor antigens observed in the periphery of anti-CTLA-4 treated patients suggests this systemic response to be at least in part tumor specific [23, 51, 52]. This bulk lymphocyte expansion in the periphery of anti-CTLA-4 treated patients translates in an on-treatment increase in absolute lymphocyte count (ALC), which correlates with improved overall survival and/or response to ipilimumab in several studies [53–56], albeit not in another [57]. Molecular imagery allowing for the mapping of cell proliferation identified secondary lymphoid tissues as the main site of lymphocyte proliferation following anti-CTLA-4 treatment, with no signals of significant proliferation in melanoma lesions themselves [58]. This is in line with the results of a study reporting on matched pre and on-treatment tumor biopsies from patients under anti-CTLA-4 revealing no change in Ki-67 expression in post-treatment TILs having expanded under therapy [30]. Effector T cells expanding in the TME under anti-CTLA-4 are therefore proposed to consist mostly of newly infiltrated clones issued from a proliferative process taking place in the periphery. Collectively, these observations highlight the importance of a systemic immune reinvigoration in the mediation of anti-CTLA-4 action on the tumor.

Observations based on the changes in TCR repertoire in the periphery under anti-CTLA-4 treatment provide contrasting information with regards to the anti-tumoral specificity of the systemic T cell reinvigoration induced by these agents. The distribution of the TCR repertoire can be described by different metrics. Its richness refers to the number of unique T cell clones represented, whereas its clonality or evenness defines the distribution of their respective frequencies. Pre and on-therapy TCR V-beta CDR3 sequencing of peripheral blood mononuclear cells (PBMC) under anti-CTLA-4 reveals an increase in richness of the TCR repertoire under therapy [59, 60]. This increase in the number of unique T cell clones under CTLA-4 blockade results from a substantial remodeling process peaking at 2 weeks after treatment start [61], involving both expansions and losses of clonotypes [60]. The evenness of the TCR repertoire under anti-CTLA-4 therapy is comparatively less affected, appearing to be either non-impacted or mildly reduced by CTLA-4 blockade [59, 61, 62]. The increase in richness of the TCR repertoire in the periphery of anti-CTLA-4 treated patients, suggested to result from unleashed T-cell priming, could be expected to enhance immune control of the tumor through the generation of new T cell responses covering a broader range of neoantigens [63]. The observation of an on-treatment increase in neoantigens detected by circulating CD8 + T cells under ipilimumab in a cohort of melanoma patients is in line with this hypothesis [64]. The polyclonal activation of T cells in the periphery induced by anti-CTLA-4 was however shown to correlate with treatment-related auto-immune toxicity rather than with tumor response in both preclinical models [33] and human studies [59, 61]. The on-treatment increase in TCR repertoire clonality in the periphery of anti-CTLA-4 treated patients is similarly biased towards immune-related adverse event (irAE) as opposed to response [62, 65]. Postow et al. observed the baseline peripheral blood TCR repertoire of melanoma patients treated by ipilimumab to be more diverse in responding patients compared to non-responders [66]. Furthermore, Cha et al. found that the maintenance of clones present in high frequency at baseline and the low rate of clonotype loss under therapy correlates with improved overall survival of a melanoma patient cohort treated by anti-CTLA-4 [60]. Together, these findings suggest the therapeutic action of anti-CTLA-4 to rest mainly on pre-existing anti-tumor reactivity and to occur despite the on-treatment remodeling of the peripheral TCR repertoire rather than as a result of it. Moreover, in a study on a cohort of early breast cancer patients treated in the neoadjuvant setting with a single dose of ipilimumab, cryoablation or a combination of both, intratumoral and peripheral T cell clones expanding under therapy were found to be poorly correlated [67]. The apparent uncoupling of the dynamics of the peripheral TCR repertoire with tumor response therefore questions the role of the T cell reinvigoration induced by CTLA-4 blockade in the periphery in leveraging the anti-tumoral action of the treatment.

## Immune suppressive T cells under anti-CTLA-4

**Fig. 1:**
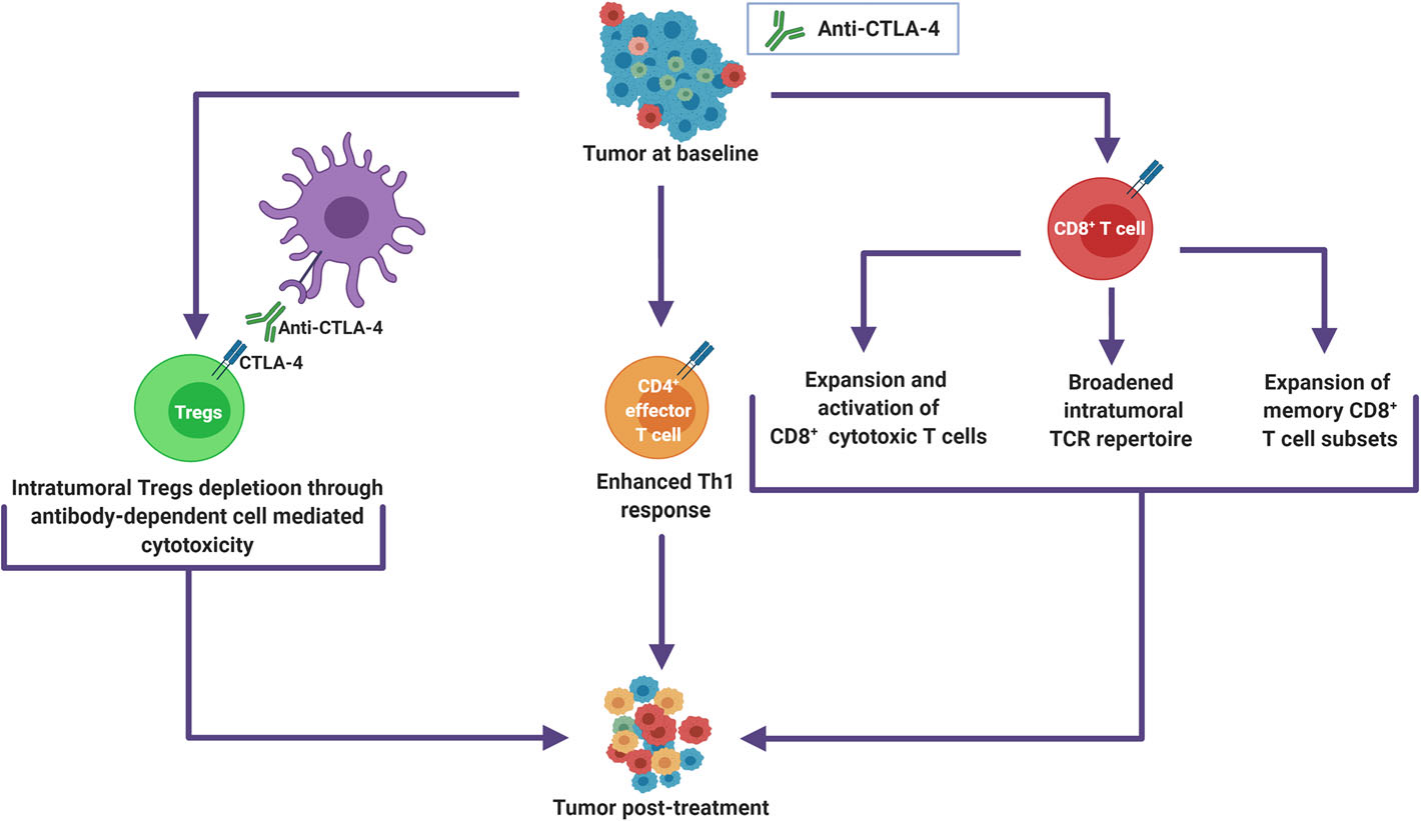
Correlation of T cell subset dynamics with the anti-tumoral action of CTLA-4 blockade in the tumor microenvironment (TME). The action of anti-CTLA-4 on the tumor has been associated with its ability to expand and activate intratumoral CD8^+^ effector T cells, broaden the range of neo-antigens targeted and induce memory CD8^+^ T cell subsets. CD4^+^ effector T cell subsets may also markedly expand in the TME on therapy and contribute to the modulation of the anti-tumoral immune response, a Th-1 response profile in the TME standing as a correlate of response to anti-CTLA-4. Another proposed, yet still debated, mechanism of anti-CTLA-4 action on the tumor is the depletion of intratumoral Tregs via a mechanism of antibody-dependent cell mediated cytotoxicity that is independent of immune checkpoint inhibition

Regulatory T cells (Tregs) are an immunosuppressive subset of forkhead box P3 (Foxp-3^+^) CD4^+^ CD25^high^ T cells known to be determinant in the regulation of the immune response to cancer [68–70]. Tregs are known to constitutively express CTLA-4 [46, 71, 72], which is a target gene of the Foxp3 transcription factor[73]. The cell-extrinsic action exerted by CTLA-4 expressed on Tregs is important to their function as mediators of peripheral immune tolerance [74, 75]. Observations issued from animal models suggest CTLA-4 could also act as a negative regulator of Treg function in a cell-intrinsic way [76]. In tumor bearing murine models exposed to anti-CTLA-4, Tregs are found to expand in the periphery [77–79], whilst simultaneously being depleted in the TME. This anti-CTLA-4 induced Treg depletion in the TME is suggested to be mediated by Fc-gamma-receptor expressing macrophages via a mechanism of antibody dependent cell-mediated cytotoxicity (ADCC) [78, 80–83], the dual action of anti-CTLA-4 on intratumoral and peripheral Tregs resting on the higher expression of CTLA-4 on exhausted tumor-infiltrating Tregs [84]. Whilst the action of anti-CTLA-4 on effector T cells (Teffs) appears to be mandatory for immune control of the tumor, therapeutic leverage of CTLA-4 blockade on TME Tregs allows for deeper responses than those observed under blockade of CTLA-4 on Teffs alone [77]. In line with this, an on-treatment increase in intratumoral Teff:Treg ratio conditions optimal immune control of the tumor under anti-CTLA-4 in a number of models [77, 78, 80, 85]. A recent study on different murine models however reports the therapeutic activity of human IgG1 anti-CTLA-4 ipilimumab to rest predominantly on Fc-dependent Tregs depletion and to be independent of checkpoint blockade [86]. The existence and relevance of this suggested mechanism of anti-CTLA-4 action remains unconfirmed in humans, where observations of on-treatment Tregs dynamics are marked by discrepancy. The expansion of Tregs in the peripheral blood of anti-CTLA-4 treated patients was documented in a number of studies [46, 87–89]. Others have however reported declining or unchanged levels of peripheral Tregs under therapy [29, 44, 49]. The predictive insight provided by the dynamics of peripheral Tregs under CTLA-4 blockade is also unclear, their on-treatment change in frequency correlating negatively [90], not at all [53, 57, 91], or even surprisingly positively [88] with anti-CTLA-4 treatment benefit in different clinical studies. More relevant to treatment outcome is the dynamics of Tregs in the TME, where Fc-dependent depletion is suggested to occur. Reports on the dynamics of intra-tumoral Tregs under anti-CTLA-4 are here again discordant. In a cohort of regionally advanced melanoma patients treated with 2 neoadjuvant doses of ipilimumab, Tarhini et al. found a trend in an inverse association between the change in on-treatment intratumoral Tregs frequency and clinical benefit [88]. Romano et al. similarly found a significant decline in intratumoral Tregs frequencies relative to total intratumoral T cells in melanoma patients responding to ipilimumab, but not in non-responding patients [92]. Two studies however report increasing levels of Tregs in on-treatment biopsies of patients treated by tremelimumab [25, 93]. Although these results could be attributed to the poor binding of this IgG2 anti-CTLA-4 subtype to the human Fc-gamma receptor, accounting for its low predicted ADCC activity, Sharma et al. also report increased frequencies of intratumoral Tregs in on-treatment biopsies of melanoma, bladder cancer and prostate cancer patients treated with the IgG1 anti-CTLA-4 ipilimumab (known to bind to most Fc human receptors), when compared to stage-matched pre-treatment biopsy controls [93]. These results do not allow to settle the debate on the impact of ipilimumab on intratumoral Tregs dynamics in humans [94]. Figure 1 illustrates T cell subsets whose dynamics in the TME under treatment were shown to correlate with anti-CTLA-4 treatment outcome. Identified dynamic biomarkers of anti-CTLA-4 treatment outcome are listed in Table 1.

**Table 1:** Dynamic immune correlates of anti-CTLA-4 and anti-PD-1 treatment outcome in clinical studies

Dynamic biomarkers	Associated outcome	Cancer type	ICI treatment	References
Expansion and activation of CD8 + effector T cell in the tumor	Clinical benefit	Melanoma	Anti-CTLA-4 monotherapy	[24, 25]
			Anti-PD-1 monotherapy	[95–97]
Increase in absolute lymphocyte count in the periphery	Clinical benefit	Melanoma	Anti-CTLA-4 monotherapy	[53–56]
Expansion in CD8 + memory T cells in the periphery	Clinical benefit	Melanoma	Anti-CTLA-4 monotherapy	[28, 29]
High on-treatment CD38 + PD-1 + CD8 + T cell counts in tumor and periphery	Progressive disease	Melanoma	Anti-PD-1 monotherapy	[98]
Expansion in CD8 + T cells in the tumor	Clinical benefit	Melanoma	Anti-PD-1 monotherapy	[97]
Upregulation of genes associated with T cell activation & T cell homing in the tumor	Clinical benefit	Melanoma	Anti-PD-1 monotherapy	[24]
Increase in INF-gamma induced transcripts in the tumor	Clinical benefit	Melanoma	Anti-CTLA-4 monotherapy	[26]
Upregulation of genes involved in antigen presentation in the tumor	Clinical benefit	Melanoma	Anti-PD-1 monotherapy	[24, 99]
Proliferative response of PD-1+/CD8 + T cells in the periphery	Clinical benefit	Multiple cancer types	Anti-PD-1 monotherapy	[100–102]
Expansion of ICOS + CD4 + T cells with Th1 like effector phenotype in the periphery	Clinical benefit	Multiple cancer types	Anti-CTLA-4 monotherapy	[40, 41]
Expansion in Tregs in the tumor	Hyperprogressive disease	Gastric adenocarcinoma	Anti-PD-1 monotherapy	[103]
Depletion of Tregs in the tumor	Clinical benefit	Gastric adenocarcinoma	Anti-PD-1 monotherapy	[103]
		Melanoma	Anti-CTLA-4 monotherapy	[88, 92]
Expansion of Tregs in the periphery	Disease progression	Melanoma	Anti-CTLA-4 monotherapy	[90]
	Clinical benefit	Melanoma	Anti-CTLA-4 monotherapy	[88]
			Anti-PD-1 monotherapy	[104]
	None	Multiple cancer types	Anti-CTLA-4 monotherapy	[53, 57, 91]
Decline in 4PD1Hi cells in the tumor or in the periphery	Clinical benefit	Melanoma	Anti-PD-1 monotherapy	[105]
Increased richness of the TCR repertoire in the periphery	irAE	Melanoma and mCRPC	Anti-CTLA-4 monotherapy	[59, 61]
Increased clonality of the TCR repertoire in the periphery	irAE	Melanoma and mCRPC	Anti-CTLA-4 monotherapy	[62, 65]
	Clinical Benefit	Multiple tumor types	Anti-PD-1 monotherapy	[106–108]
Depletion in specific MDSCs subsets in the periphery	Clinical benefit	Melanoma		[88, 109]
Decline in bulk B cells coinciding with an increase in CD21^lo^ B cells or plasmablasts in the periphery	irAE	Melanoma	Combined anti-CTLA-4/ anti-PD-1	[110]

*Trend in association, not reaching statistical significance (*P* = 0.09)

## Effector T cells under anti-PD-1

When engaged to its PD-L1 and PD-L2, PD-1 inhibits downstream signaling of the TCR, thereby negatively regulating T cell activity [111]. PD-1 expression is induced on T cells upon their activation, whilst its ligands can be expressed by a range of cell types, including tumor cells and other non-immune cell subsets, in response to inflammatory cytokines [112]. PD-1/PD-L1 inhibitors are understood to act by interfering with this negative feedback loop regulating T cell activation. In line with this hypothesis, the upregulation of genes associated with enhanced effector T cell activity stands as a consistent correlate of response to these agents [24, 95, 106]. Matched pre and on-treatment biopsies issued from clinical studies show the action of PD-1 blockade on the tumor to translate into a marked intratumoral expansion of CD8 + effector T cells coinciding with a change in gradient of CD8 + T cell density from the tumor margins into its center [95–97]. This on-treatment expansion in CD8 + T cells coincides with an increase in CD8 + T cell clonality in the TME [96], suggesting that the intratumoral CD8 + T cells expanded on therapy are tumor-reactive.

There is considerable reported heterogeneity in phenotypes and functional states amongst tumor-infiltrating CD8 + T cells. A subset of interest expresses high levels of PD-1 and co-express Tim-3, lymphocyte-activation protein 3 (LAG3), T cell immunoreceptor with Ig and ITIM domains (TIGIT) and CD39, defining a phenotype of exhaustion (Tex) [113, 114]. Exhausted CD8 + T cells in the TME have a higher potential for tumor antigen recognition and have a higher clonal distribution than any other CD8 + T cell subsets represented in the TME [115, 116], in line with the understanding that this dysfunctional cell state develops as a function of chronic antigen exposition. The Tex phenotype translates functionally into an impaired capacity in IL-2, tumor necrosis factor alpha (TNF-α) and IFN-γ effector cytokine production [115]. PD-1 blockade has been suggested to reinvigorate the immune response against the tumor by reversing the state of terminally exhausted tumor-antigen-experienced CD8 + T cells. This paradigm is however challenged by the observation that the terminal state of exhaustion is associated with a distinct epigenetic profile limiting cell-state reversibility [117–119]. Recent evidence suggests that anti-PD-1 engages progenitor Tex subsets co-expressing PD-1 and tcf-1 rather than terminally exhausted subsets [120, 121]. In animal models, this engagement of anti-PD-1 on PD-1+/tcf-1 + CD8 + T cells induces their self-regeneration or their further differentiation into terminal effector cells, losing T cell factor 1 (tcf-1) expression and developing functional states of exhaustion in the process [122, 123]. This is in line with the observation that anti-PD-1 associates with an increase, rather than a decline, in intratumoral CD8 + T cells displaying phenotypes of terminal exhaustion [124, 125]. Of note, tumor infiltrating Tex subsets preserve their capacity to proliferate until terminal exhaustion states [116, 126] and were shown to play an active role in the recruitment of other immune subsets to the TME through secretion of chemokine ligand 13 (CXCL13) in non-small cell lung carcinoma (NSCLC) tumors [115]. This suggests that Tex may play an active role in the anti-tumoral immune response. Anti-PD-1 was also shown to engage distinct memory-precursor like CD8 + T cell subsets, leading to their accumulation in the tumor [127]. The on-treatment intratumoral accumulation of CD8 + T cells with memory-like features has been associated with enhanced cytotoxicity [128] and stands as a correlate of response to PD-1 blockade in preclinical models as well as in clinical studies [97, 129], suggesting this to be an important pathway mediating anti-PD-1 tumor control. These observations collectively show that PD-1 blockade promotes the anti-tumoral CD8 + T cell response by engaging distinct Tex progenitor subsets, rather than by reversing the state of terminally exhausted T cells.

Genes related to antigen presentation, T cell activation and T cell homing are amongst the most highly upregulated in tumors responding to PD-1 blockade [24]. In paired pre and on-treatment biopsies of basal cell carcinoma patients treated by nivolumab, a significant proportion of specific TCR sequences of clonally expanded tumor-infiltrating Tex post-treatment were not detected in site-matched pre-treatment biopsies [124]. This corroborates the documentation of new neoantigen-specific T cell responses in the TME of matched pre and post-treatment biopsies of melanoma tumors following 2 doses of neoadjuvant nivolumab [130]. Yost et al. report that a significant fraction of the TCR sequences of clonally expanded CD8 + T cells in post anti-PD-1 treatment biopsies were absent in baseline tumors whilst present in the peripheral blood of treated patients before and on-treatment. The CD8 + effector T cell response induced by anti-PD-1 may therefore in part rely on the influx of new CD8 + T cell clones issued from the periphery, in line with preclinical models showing impaired response to anti-PD-1 when interfering with the migration of immune cells into the tumor [129].

The systemic response to PD-1 blockade manifests as an increase in the proliferation of peripheral CD8 + T cells early into the course of anti-PD-1 therapy in treated patients [100, 125]. It coincides with dynamic changes in the distribution of the peripheral TCR repertoire, PD-1 blockade impacting on its evenness more than its richness [96, 106, 107]. This on-treatment increase in clonality of the peripheral TCR repertoire correlates with treatment benefit in a number of studies [106–108], as does a high frequency of shared clones between the peripheral blood and the TME [124]. We have recently observed the frequency of peripheral CD107^+^ CD8^+^ cytotoxic T cells directed against a known cancer testis antigen in repeated longitudinal peripheral blood sampling of a gastric cancer patient treated by an anti-PD-1 to closely correlate with different phases of disease evolution under treatment [131]. These observations suggest the systemic CD8 + effector T cell response to be an important leverage of anti-PD-1 tumor control. A number of studies have investigated the CD8 + T cell proliferative response in the peripheral blood as a predictive biomarker of anti-PD-1 benefit. Analyzing pre and on-treatment peripheral blood samples of stage IV melanoma patients treated with pembrolizumab, Huang et al. [100] report the on- treatment fold change in Ki67^+^/PD-1^+^ CD8^+^ T cells by 6 weeks of first dose of anti-PD-1 administration to correlate with objective response rate, progression free survival and overall survival after adjusting for a measure of tumor burden at baseline. The timing of the proliferative response was shown to impact on the predictive ability of this biomarker in a study on NSCLC patients treated with pembrolizumab, where the PD-1^+^ CD8^+^ T cell proliferative response after a single dose of a PD-1 inhibitor was found to correlate with response only if it occurred within 4 weeks of treatment initiation [101]. Further study into the early dynamics of circulating PD-1^+^ CD8^+^ T cells revealed the peak of the proliferative response to occur as early as 7 days following the first dose of anti-PD-1 [102, 125]. The fold increase in PD-1^+^/Ki67^+^ CD8^+^ T cells from baseline to 7 days after the first pembrolizumab administration predicted durable clinical benefit (DCB) with a sensitivity and specificity of 90 % and 75 % respectively, in thymic epithelial tumor patients [102].

In contrast with anti-CTLA-4, PD-1 blockade exerts a limited impact on the on-treatment dynamics of tumor infiltrating CD4 + effector T cells in murine models [19]. The study of matched pre/on-treatment biopsies of melanoma patients having received an anti-PD-1 revealed a decrease in frequency of effector memory CD4 + T cells post-treatment, whilst an on-treatment increase in intratumoral CD4 + effector T cells was found to be a negative correlate of response [97]. The role of CD4 + effector T cells, if any, in mediating anti-PD-1 tumor control, is to date therefore not established.

## Immune suppressive T cells under anti-PD-1

Tregs can express varying levels of PD-1 and PD-L1, a comparatively higher expression of PD-1 being found in those infiltrating the tumor [132–134]. The PD-1/PD-L1 axis is an important pathway mediating the inhibition of Teffs by Tregs in murine tumors, chronic infection or autoimmunity models, where this inhibition is demonstrated to be largely mediated by direct cell contact [135–138]. PD-1 blockade could therefore contribute to the anti-tumoral immune response by interfering with this *trans* inhibitory mechanism of Teffs function. The PD-1/PD-L1 axis however also modulates Tregs function via cell-intrinsic pathways. PD-1 blockade translates into a reduced immunosuppressive function of Tregs and their decline in the TME in a number of preclinical murine model studies [135, 139]. These results are corroborated by an *in vitro* study based on PBMCs obtained from advanced melanoma patients, where anti-PD-1 was found to induce resistance of cytotoxic T cells to Tregs inhibition, to reduce the immunosuppressive function of Tregs and to result in their down-regulation of Foxp3 [140]. In murine models, it has been shown that the PD-1/PD-L1 axis mediates the conversion of CD4 + Th1 effector T cells into induced Foxp3 + regulatory T cells (iTregs) [141, 142] and sustains iTregs function by contributing to maintain their Foxp3 expression [142–144]. Other preclinical studies however show PD-1 blockade to correlate with an increase rather than a decline in Tregs infiltration in the TME [145]. An increase in intratumoral proliferation of Tregs observed after a single dose of neoadjuvant pembrolizumab correlated inversely with the recurrence-free survival of a melanoma patient cohort [125]. Although the mechanism underlying such a PD-1 induced proliferative surge in Tregs in the tumor are not clearly established, the possible contribution of a counter-regulatory feedback mechanism in response to a re-invigorated CD8 T cell response is plausible. A direct induction of Tregs proliferation by anti-PD-1/PD-L1 may however also come at play. PD-1-Hi Tregs resident in human glioblastoma tumors were found to be dysfunctional and to express genes enriched in exhaustion signatures [133]. Exhausted PD-1-Hi Tregs subsets obtained from chronic infection contextures display enhanced proliferation under PD-L1 blockade both *in vitro* [146] and *in vivo* [147], suggesting that anti-PD-L1 have the capacity to rescue Tregs in the exhausted cell-state. In a chronic lymphocytic choriomeningitis virus (LCMV) model study, anti-PD-L1 allowed the rescue of exhausted CD8 + T cells early into the course of infection but failed to do so in its later stages, where it resulted instead in the substantial expansion of PD-1^+^ Tregs [147]. This paradoxal effect of PD-1/PD-L1 blockade is reminiscent of the marked infiltration by highly proliferative Foxp-3^Hi^/CD45^−^ CD4^+^ T cells (effector Tregs) reported in biopsies of gastric adenocarcinoma patients presenting with hyperprogressive disease under anti-PD-1 treatment which contrasted with responders who displayed a decline in intratumoral Tregs frequencies upon treatment [103]. An expansion of Tregs can be observed in the peripheral blood of patients early into the course of anti-PD-1 therapy [104, 148]. This expansion in circulating Tregs correlated with a reduction in their immunosuppressive function as well as with disease non-recurrence, when observed in the peripheral blood of resected melanoma patients treated by adjuvant nivolumab therapy [104]. Further study into the dynamics of circulating Tregs under PD-1 blockade is necessary to assess their functional relevance and predictive value. These observations collectively suggest the action of PD-1 blockade on Tregs could have both positive and detrimental effects on the immune response to cancer. This latter point serves as a rational for ongoing studies into the benefit of combining PD-1/PD-L1 blockade with agents impacting on the TGF-beta signaling pathway [145, 149].

**Fig. 2:**
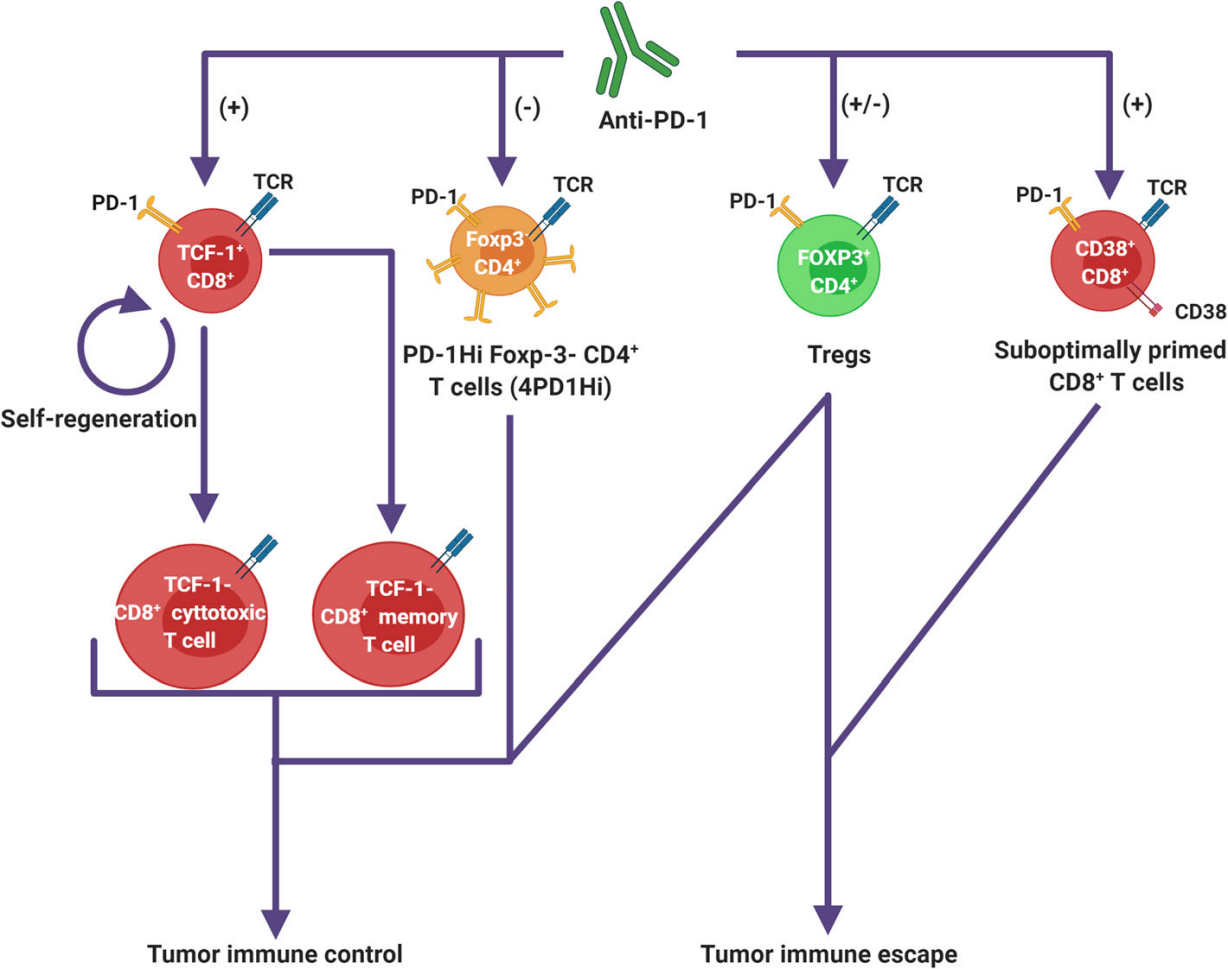
Identified T-cell subset pathways modulating the action of anti-PD-1 on the tumor. Anti-PD-1 antibodies are suggested to engage distinct transcription factor T cell factor 1 (TCF-1) expressing precursor CD8^+^ T cell subsets, thereby inducing either their self-regeneration or their further differentiation into terminal effector CD8^+^ T cells. Enhanced tumor infiltration by cytotoxic T cells and CD8^+^ memory T cells both stand as correlates of an effective re-invigoration of the anti-tumoral immune response under PD-1 blockade. Anti-PD-1 can also directly engage an immune suppressive Foxp3^−^ CD4^+^ T cell subset expressing high levels of PD-1 and displaying a T follicular-helper profile (4PD1Hi) cells, inducing their downregulation in the tumor microenvironment and in the periphery. Anti-PD-1 may also contribute to anti-tumoral immunity by infringing the inhibition of effector T cells by PD-1^+^ Tregs in the TME mediated by direct cell contact (*trans* mechanism). PD-1 blockade also induces potentially opposite *cis*-effects on PD-1^+^ Tregs, leading either to their downregulation (reduced immune suppressive function and downregulating Foxp-3 expression) or to their expansion (through the reversal of a functional state of exhaustion). Anti-PD-1 antibodies may induce the expansion of immune suppressive CD8 + T cell subsets, including that of a CD38^+^ PD-1^+^ CD8^+^ T cell subset suggested to result from suboptimal priming, which will have an adverse influence on the anti-cancer immune response

Another immunosuppressive CD4 + T cell subset found to be regulated by anti-PD-1 has recently been identified. These cells, referred to as 4PD1^Hi^, express high levels of PD-1, lack Foxp-3 expression and are further characterized by a T-Follicular Helper profile [105]. 4PD1^Hi^ cells were shown to accumulate in the tumor as a function of tumor progression and were shown to exert a direct inhibition on T cell effector function. CTLA-4 inhibition was shown to induce tumor infiltrating and circulating 4PD1^Hi^ cells, whereas anti-PD-1 treatment exerted an opposite effect on this cell subset. Downregulation of tumor-infiltrating and circulating 4PD1^Hi^ populations under anti-PD-1 treatment was further documented as a correlate of response to pembrolizumab in a melanoma patient cohort. Specific subsets of CD8 + T cells expanding under anti-PD-1 were also found to correlate positively with tumor growth, suggesting their immunosuppressive role [19]. An immunosuppressive CD8 + T cell subset coexpressing PD-1 and CD38 expanding upon anti-PD-1 therapy under conditions of suboptimal priming, has recently been identified [98]. In post treatment biopsies of anti-PD-1 treated melanoma patients, a percentage of PD-1^+^/CD38^+^ cells > 4 % amongst the total CD8 + T cell population predicted treatment failure (p < 0.001). Furthermore, the on-treatment dynamics of the PD-1^+^/CD38^+^ CD8 + T cell population in the peripheral blood was also predictive of therapeutic outcome. Identified T cell subsets mediating anti-PD-1 action on the tumor are depicted in Fig. 2. Identified dynamic biomarkers of anti-PD-1 treatment outcomes are listed in Table 1.

## B cells under CTLA-4 and PD-1 blockade

The immune response to cancer has been mostly studied under the scope of the T cell compartment. B cells, which represent 25–33 % of the immune infiltrate of human melanoma tumors [150, 151], are however increasingly recognized as important actors and regulators of the anti-tumoral immune response. Tumor associated B cells (TAB) display a wide range of phenotypes and functions [152]. TAB may secrete pro-inflammatory cytokines and may generate antibodies possibly mediating antibody tagged tumor-cell death through complement activation and opsonization. TAB may also contribute to T cell mediated tumor death through their capacity to function as potent antigen presenting cells [153, 154]. TAB may however also exert an immunosuppressive effect, as depicted by so-called B regulatory (Bregs) subsets, notably through the production of cytokines such as IL-10 and transforming growth factor beta (TGF-β) [155]. Wang et al. notably identified a B-cell subset expressing PD-1 in differentiated thyroid tumors, capable of functionally suppressing T cell activity [156]. This duality in action may account for the conflicting results of animal model studies with regards to the role of B cells in the anti-tumoral immune response, as well as for the divergence in the reported prognostic insight provided by the degree of B cell infiltration in different tumor subtypes [157]. The intratumoral B cell infiltration nonetheless consistently correlates with prolonged patient survival when associated with the presence of tertiary lymphoid structures (TLS) within tumor tissue. TLS have been documented in a range of solid tumor types as well as in autoimmune disease and transplanted organs, where they stand as surrogate markers of immune activation [158, 159]. Several studies report TLS-associated gene-expression signatures in tumors at baseline to be predictive of longer overall survival in patients treated with PD-1 and/or CTLA-4 blockade [150, 160, 161]. This prognostic ability could be independent of the therapeutic impact of immune checkpoint therapy. The presence of TLS within a tumor could also be the surrogate of an active and chronicized anti-tumoral immune response, thereby signaling a potentially favorable terrain for ICI action. There is however growing evidence suggesting an active role of B cells and TLS in the anti-tumoral immune response generated by anti-CTLA-4 and anti-PD-1 treatment.

Using murine models of triple negative breast cancer treated by combined PD-1 and CTLA-4 blockade, Hollern et al. found response to therapy to correlate with a significant elevation in the intratumoral expression of a B cell mRNA signature [162]. In these models, the on-treatment expansion in tumor-infiltrating B cells coincided with increased expression of markers associated with B cell activation and proliferation in class-switched B cells and increased expression of major histocompatibility complex (MHC) class II genes in non-classed switched cells. Interestingly, the selective ablation of B cells within the tumor abrogated therapy response and resulted in lower on-treatment CD4 + and CD8 + T cell tumor infiltration along with a lower representation of effector memory CD8 + T cell subsets. Inversely, the intratumoral presence of T cells was also conditional to therapy induced B cell activation, which in this study was found to rest on a CD4 + follicular helper T cell subset induced under treatment. The importance of the cooperation of follicular helper T cells with Th1 cells and B cells in orchestrating the anti-cancer immune response is corroborated by their association with a favorable prognosis in breast and colon cancer [163, 164]. Additional evidence issued from longitudinal sample collection issued from patients under ICI supports a role for B cells in leveraging the anti-tumoral action of ICI treatment. Cabrita et al. describe a gene expression signature associated with the presence of TLS in melanoma tumors to correlate with response to PD-1 blockade when applied to on-treatment tumor biopsies of two independent melanoma patient cohorts. Interestingly, this TLS gene expression signature was in contrast not predictive of response in pre-treatment biopsies of the same patients. In another study, post-treatment intratumoral B cell counts and B cell associated gene signatures strongly correlated with response to anti-PD-1 +/- anti-CTLA-4 in locally advanced melanoma patients exposed to neoadjuvant ICI therapy, whilst B cell counts at baseline did not [165]. Preliminary evidence therefore suggests a possible role of TAB in shaping T cell response and mediating the anti-tumoral action of ICI therapy, although much remains unknown in this emerging field of research. It is also unclear whether this potential ICI-induced effect on B cells could be registered in the periphery. In a study reporting on B cell dynamics in the peripheral blood of melanoma patients under anti-CTLA-4, anti-PD-1 or their combination, Das et al. reported that combined therapy correlated with a decline in circulating B cells coinciding with an increase in CD21^lo^ B cells and plasmablasts detectable within a single cycle of therapy [110]. Interestingly, these changes did not correlate with treatment benefit, but were instead found to be associated with increased rates of grade III or higher irAE within 6 months of therapy.

## Myeloid cell compartment under CTLA-4 and PD-1 blockade

Monocytes, macrophages and dendritic cells are involved in antigen presentation and T-cell priming and are as such key constituents of the cancer immunity cycle, bridging the innate to the adaptive immune response. Cancer related chronic inflammation however disturbs the myeloid cell line maturation process, leading to the development of myeloid derived suppressor cells (MDSCs) and tumor-associated macrophages (TAMs) which are potent suppressors of the anti-tumoral immune response [166]. Tumor associated monocytes and macrophages display a wide range of phenotypes and function, spanning across two ends of a spectrum represented by pro-inflammatory (M1) and immunosuppressive (M2) extremes [167]. Several studies in animal models suggest the possibility for ICI treatment to induce a striking remodeling of the intratumoral myeloid cell compartment from an immunosuppressive composition to a pro-inflammatory one [168, 169]. Post-treatment TAMs display an increased expression of MHC and of co-stimulatory molecules [169]. Such reprogramming of TAMs may contribute to anti-tumoral T cell responses through *in situ* antigen presentation [170], possibly accounting for the enhanced effector T cell response this ICI induced myeloid remodeling was shown to convey [169].

**Fig. 3:**
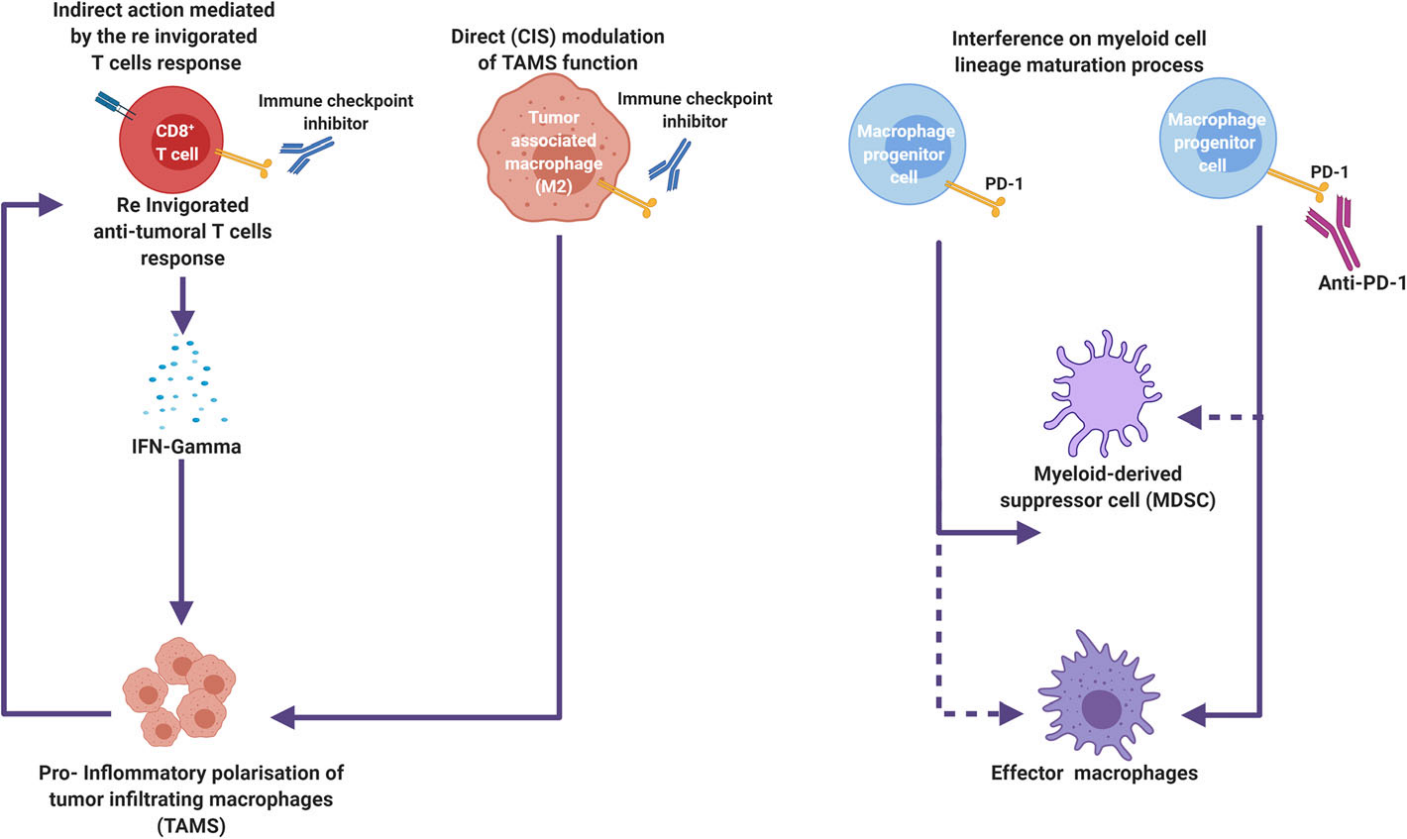
Proposed mechanisms for the reconfiguration of tumor-associated macrophages under immune checkpoint blockade. Both CTLA-4 and PD-1 blockade have shown to be capable of inducing a profound remodeling of the myeloid cell compartment in the tumor and in the periphery, reconfiguring its immunosuppressive function into a pro-inflammatory one. This effect has been suggested to be mediated indirectly, via the reinvigoration of T cells and the INF-gamma cytokine secretion allowing for the pro-inflammatory polarization of monocytes newly infiltrating the tumor. A direct *cis*-action of anti-PD-1 and anti-CTLA-4 on tumor-associated macrophages expressing these receptors, resulting in part in reduced arginase-1 expression, is also proposed. This reconfiguration of the intratumoral myeloid compartment may in turn contribute to enhance the T cell response directed against the tumor notably through the capacity of tumor-associated macrophages to act as potent antigen-presenting cells. PD-1 blockade was also shown to impact on the myeloid cell lineage maturation process, allowing myeloid precursor cells to pursue their maturation into terminal effector macrophages, thereby overcoming the block in maturation leading to the generation of MDSCs observed in cancer-associated emergency myelopoiesis

INF-gamma secreted by reinvigorated T cells is a possible indirect mediator of this ICI-induced myeloid cell reprogramming in the TME [168]. In another preclinical study, dual PD-1 and CTLA-4 blockade was found to induce an increase in pro-inflammatory macrophages in the TME of animal models via a mechanism involving a specific subset of Foxp3^−^ CD4^+^ T cells [171]. Direct mechanisms of regulation of MDSCs by PD-1 or CTLA-4 blockade were also identified. CTLA-4 is expressed on human monocytes and can be induced on monocyte-derived dendritic cells (mDC), where it acts as a negative regulator of mDC cytokine secretion and of mDC-associated antigen-specific CD4 + T cell proliferation [172]. In mice, subsets of tumor-derived MDSCs expressing PD-1 and CTLA-4 display decreased arginase 1 expression and activity upon CTLA-4 or PD-1 blockade *in vitro* [173]. This accounts for a possible mechanism contributing to anti-CTLA-4 and anti-PD-1 anti-tumoral action, arginase 1 activity impairing T cell function in the TME of murine models, thereby contributing to immune evasion [174]. Furthermore, PD-1 blockade was recently shown to interfere with the maturation block of cells of myeloid lineage resulting in the accumulation of MDSCs under conditions of emergency myelopoiesis. This allowed the maturation of myeloid precursors to proceed into effector macrophages and dendritic cells, thereby contributing to reorganize the myeloid composition of the TME into one contributing favorably to the immune response [175]. Interestingly, myeloid-specific PD-1 ablation lead to a greater impact on tumor control than T cell-specific PD-1 ablation in this model. These proposed mechanisms of ICI action on the myeloid cell compartment are depicted in Fig. 3. These preclinical observations suggest immune checkpoint inhibitors to be capable of influencing the composition of the intratumoral myeloid cell compartment, whether in a direct or in an indirect way. This may however be insufficient to overcome the immunosuppressive action exerted by the higher macrophage intratumoral densities found in late stages of tumor evolution [169]. Several preclinical studies demonstrated improved tumor control through the combination of ICI with agents depleting or functionally impairing MDSCs (which include PI3K inhibitors and entinostat amongst others) [176–180], setting the ground for ongoing clinical trials testing such combinations.

The on-treatment decline in bulk circulating MDSCs levels in given specific MDSCs subsets have been documented under anti-CTLA-4 in a number of clinical trials, where this parameter correlates, to some degree, with patient outcome [29, 48, 88, 109]. This association is however not universally reported [181]. Furthermore, the dynamics and predictive value of the main MDSCs subsets defined as monocytic MDSCs (mo-MDSCs) and polymorphonuclear MDSCs (PMN-MDSCs) based on distinct surface marker expression and function [182], were highly discrepant in these studies. Similarly, whilst the level of circulating mo-MDSC and PMN-MDSC subsets were not shown to be affected by PD-1 blockade in several clinical studies [97, 183, 184], high-dimensional single-cell analysis applied to the peripheral blood of metastatic melanoma patients treated with an anti-PD-1 revealed a striking remodeling of the myeloid compartment 12 weeks after initiation of therapy [183]. The considerable heterogeneity [167] and plasticity [185] of the myeloid cell compartment points to the necessity to study MDSCs dynamics under ICI using comprehensive cellular and molecular profiling platforms. An enhanced ability to monitor the evolution of myeloid cells under immune checkpoint blockade is critical, provided the considerable impact these may have in both facilitating, or inhibiting the effector T cell response elicited by these therapies.

## Monotherapy versus multiple checkpoint blockade

The upregulation of alternative immune checkpoints stands as a pathway of immune escape to ICI monotherapy. In murine models, blocking one immune checkpoint induces the upregulation of alternative immune checkpoints in tumor infiltrating T cells [186, 187]. The observation of significant V-domain Ig suppressor of T cell activation (VISTA) and PD-L1 upregulation in the immune infiltrate of prostate tumors after two doses of ipilimumab therapy suggests that such mechanisms of adaptive immune resistance may be induced early into therapy [188]. In this study, the increase in expression of alternative immune checkpoint in post-treatment biopsies was more important in prostate cancer tumors than in melanoma tumors exposed to the same therapy, possibly accounting for the limited benefit offered by ipilimumab in prostate cancer patients [189, 190]. These observations suggest the early upregulation of alternative immune checkpoint in the tumor immune infiltrate could convey information predictive of treatment outcome. This information could in turn be leveraged into guiding tailored therapeutic action aiming to overcome such immune resistance pathways. In a study documenting the upregulation of TIM-3 on tumors of mice having developed resistance to PD-1 blockade, the sequential addition of a TIM-3 inhibitor was shown to recover immune control of the tumor, suggesting the potential benefit of such an approach [186]. Upfront multiple checkpoint blockade stands as an alternative approach. In advanced melanoma patients, combining ipilimumab and nivolumab induces higher response rates as compared to either agent in monotherapy and translates into 5-years overall survival of 52 %, compared to 44 % and 26 % in nivolumab and ipilimumab single treatment groups, respectively[1]. Despite being associated with an increased incidence of irAE, the therapeutic benefit conferred by this combination led to the Food and Drug Administration (FDA) to grant its further approval in intermediate and poor risk advanced renal cell carcinoma, metastatic NSCLC with > 1 % PD-L1 expression as well as in hepatocellular carcinoma [3, 191, 192]. In preclinical models, the higher potency of the anti-CTLA-4/anti-PD-1 combination translates into markedly increased CD8^+^ T cells/Tregs and CD8^+^ T cells/MDSCs ratios in post-treatment biopsies compared to single checkpoint blockade [187, 193]. In murine models, the combination of anti-CTLA-4 and anti-PD-1 induces changes in gene expression that are interestingly largely non-overlapping with those observed under either agent in monotherapy [194]. Similarly, in a human study, CTLA-4 and PD-1 combined blockade was reported to induce the expression of distinct sets of genes in T cells, differing from those induced in patients under anti-PD-1 or anti-CTLA-4 alone [45]. These observations suggest that combined immune checkpoint blockade induces a pattern of immune modulation that differs from the sum of its parts.

Interestingly, different classes of ICI may also influence one-another’s action when administered sequentially, as exemplified by a prospective study on advanced melanoma patients randomized to receive nivolumab followed by ipilimumab or to these same drugs administered in an inverse order, showing higher response rates in the prior sequence as compared to the latter, along with distinct irAE profiles [195]. These clinical observations echo results of studies reporting the immune modulation exerted by ICI to be influenced by prior treatment with another ICI subclass. The expression of genes induced in peripheral T cells by PD-1 blockade is reported to be influenced by past CTLA-4 exposition status in a number of studies [24, 45, 106]. Prior treatment with an anti-CTLA-4 was also shown to impact on the predictive ability of TCR repertoire dynamics in a cohort of melanoma patients treated with an anti-PD-1 [106]. In this study, therapeutic response was shown to correlate with an increase in clonality of the TCR repertoire in ipilimumab naïve melanoma patients, whilst correlating instead with an increase in its richness in patients pre-exposed to the anti-CTLA-4. The predictive potential of CD8 + T cells expansion under PD-1 inhibition was similarly impacted by prior anti-CTLA-4 exposition, correlating with response only in ipilimumab naïve patients. CTLA-4 blockade therefore appears to leave a long-lasting print in the immune system, which may impact the immunomodulation exerted by future exposition to an anti-PD-1.

## Immunoediting and other tumoral mechanisms of immune escape

**Fig. 4:**
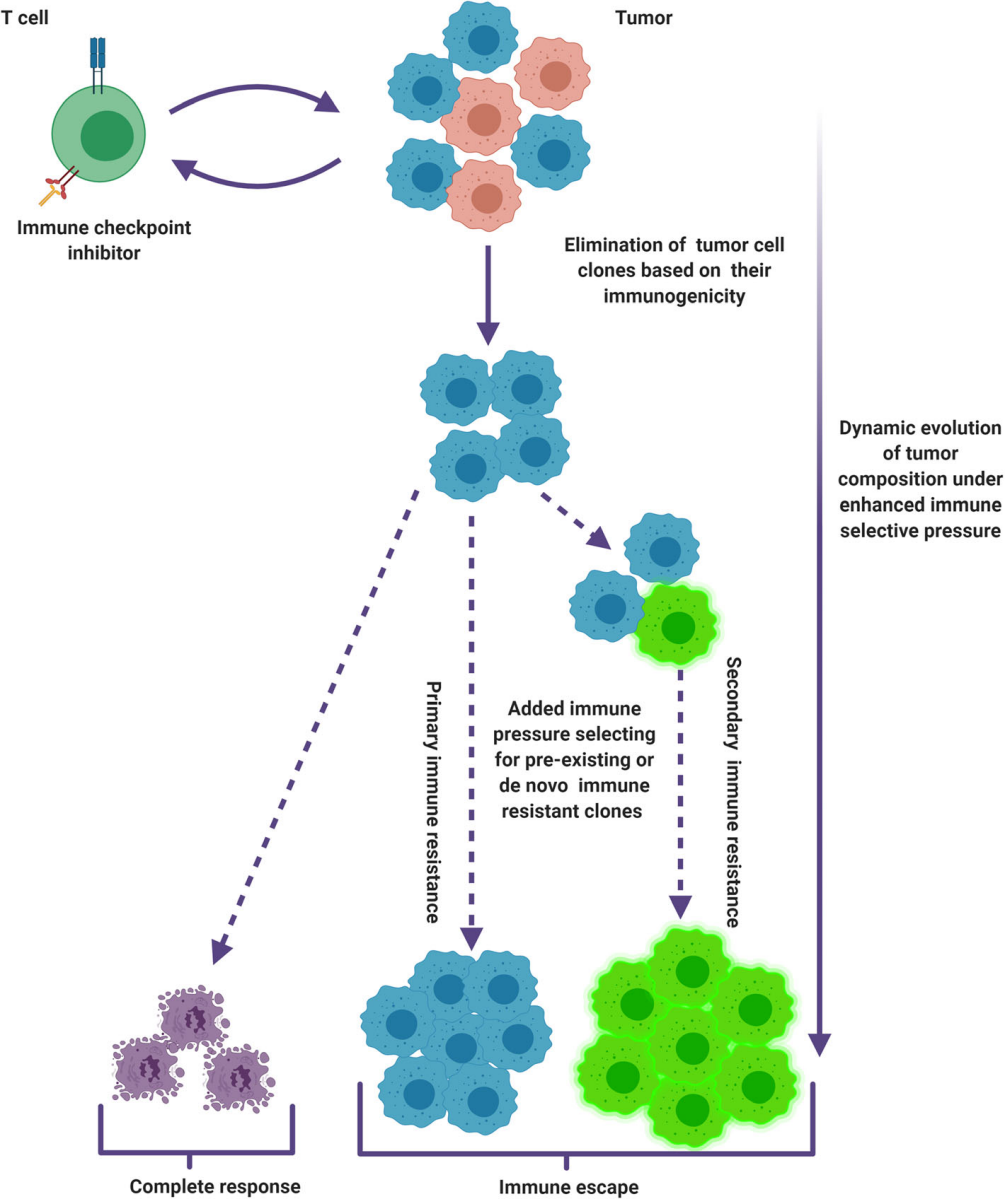
Dynamic interaction of the tumor with the anti-tumoral immune response induced by immune checkpoint blockade. The immune response directed against the tumor may induce changes in tumor cells that will participate in the regulation of this response. As, such, tumor cells are active regulators of the immune response directed against the TME. Tumor cells have the potential to both enhance or weaken the immune system (e.g. through the upregulation of genes involved in the antigen-presentation machinery in response to INF-gamma), or (e.g. through the expression of PD-L1 or the upregulation of pathways actively participating to the exclusion of T cells from the TME) respectively. The resulting immune response will in turn contribute to dynamic changes in tumor clonal composition. An effective anti-tumoral immune response will translate into the elimination of tumor cell clones as a function of their immunogenicity. This may in time select for pre-existing or *de novo* immune resistant clones that have the capacity to thrive in spite of the immune pressure induced by ICI, leading to secondary immune resistance

The indirect mode of action of ICI implies therapeutic response to rest on their ability to reconfigure the anti-tumoral immune response. This added immune pressure has been shown to induce changes in tumor composition early into the course of therapy (Fig. 4). In paired baseline and on-treatment biopsies issued from melanoma patients exposed to anti-PD-1 therapy, a high frequency of clonal variants decreasing on treatment correlates with a favorable treatment outcome [106]. Inversely, the frequency of novel single nucleotide variants (SNVs) appearing under treatment correlates with stable or progressive disease. In this study, the authors also measured changes in tumor mutational burden (TMB) from baseline to week 4 of nivolumab treatment. Interestingly, a reduction in TMB under nivolumab strongly correlated with response and was more predictive of treatment outcome than pre-treatment TMB alone. Early on-treatment changes in tumor composition may therefore serve as early signals of response. This illustrates the capacity of the immune system to infer on tumor development and subsequently shape tumor composition as part of a process referred to as immunoediting. Conversely, a lack of immunoediting early into the course of therapy may point to primary immune resistance and serve as a negative correlate of ICI treatment benefit. Theoretically, immunoediting will however in time select for lesser immunogenic clones, thereby favoring immune escape translating into secondary immune resistance [196–198].

Immune resistance can be mediated by the engagement of a number of identified pathways. Tumors displaying signatures that reflect a cytotoxic response were shown to harbor recurrent sets of specific loss of function mutations in genes involved in antigen surface expression and extrinsic apoptosis signaling pathways (such as CASP-8), along with genetic amplifications in genes involved in immune evasion (such as PD-L1/2) [199]. Mutations impacting on the function of the antigen presentation machinery stand as important determinants of tumor immunogenicity suggesting their relevance with regards to ICI benefit. Point mutations, deletions or loss of heterozygosity in beta-2-microglobulin (β-2 M) were documented in 30 % of melanoma patients progressing under ICI [200]. β-2 M is a key protein involved in the transport of MHC class I to the cell surface, loss of function mutations of the β-2 M genes providing cells with a means of escaping neoantigen recognition by T cells in the TME. The selective expansion of β-2 M mutated clones despite a successfully ICI-induced reinvigorated CD8 + T cell response has been further documented in a number of studies [106, 125, 201]. Tumor cell-intrinsic pathways suggested to actively exclude T cells from the TME have also been identified. These include phosphoinositide 3-kinase (PI3K) activation (through loss of PTEN), TP53 mutations, MYC and WNT-B-catenin signaling pathways [202], alterations in the latter notably correlating with reduced immune cell infiltration in melanoma and colorectal cancer tumors [203, 204]. Whole exome sequencing on paired baseline and progressing lesions of melanoma patients having initially responded to anti-PD-1 therapy, identified the loss of function mutations in JAK1 and JAK2 genes, which are involved in the IFN-γ signaling pathway, as mechanisms of acquired resistance to PD-1 inhibitors [201]. Defects in the INF-gamma signaling pathway were similarly shown to confer resistance to anti-CTLA-4 therapy in melanoma patients [205]. In a recent study on baseline and on-treatment tumor biopsies of a melanoma patient cohort, Grasso et al. observed the anti-tumoral action of ICI to rest in part on INF-γ induced changes in gene expression in melanoma tumor cells resulting in their increased expression of surface antigens and reduced expression of genes associated with immune exclusion [99]. INF-γ issued from the T cell response reinvigorated by ICI is therefore suggested to mediate a cross-talk between immune cells and tumor cells contributing to enhance the anti-tumoral immune response. This highlights further the need to consider ICI action under a system approach whereby tumor cells and immune cells are constitutive entities of the same system.

## Present and future perspectives on immune monitoring under ICI

The study of immune dynamics within the TME under ICI is critical to gain understanding into mechanisms mediating ICI treatment activity. However, the immune contexture may differ in different metastatic sites of the same patients, translating into distinct trajectories of immune responses in individual lesions in time [206, 207]. These observations challenge the utility of single lesion biopsies to comprehensively assess the anti-tumoral immune response against cancer in a multi-metastatic patient. Furthermore, repeated tumor tissue biopsies imply a considerable burden and risk of complications for patients. Although the extent to which immunological events occurring at the level of the TME are represented in the periphery remains to be further elucidated, preclinical and clinical observations discussed in this review suggest the therapeutic effect of ICI to be mediated, at least in part, by the systemic response they generate. As such, immune processes registered on circulating leukocyte populations could be valid surrogates of the clinical action of ICI. One must however acknowledge that some of the evoked pathways of ICI action and resistance rest on processes occurring within the TME. It is plausible that such local mechanisms may not be captured in the periphery. Although the perspective of monitoring ICI action in the peripheral blood is appealing in many regards, some information may be restricted to the TME, possibly including that underlying dissociate metastatic site-specific disease evolutions under treatment.

The findings presented in this review highlight the potential for ICI to impact on a number of immune pathways interfering with the complex and dynamic interaction of the tumor with the immune system. A restrictive focus on a limited number of immune processes is thereafter unlikely to capture the full scope of ICI action. The advent of high throughput sequencing paved the way of systems approaches, whereby all the constitutive elements of a system present in a sample are measured in a non-targeted and unbiased manner [208]. The comprehensive molecular profiling offered by such technologies is particularly appealing to the field of immune monitoring, provided the large number of cell protagonists and immune pathways that may come into play in an immune response. Transcriptomics is the measure of the abundance of all transcripts in a sample on a genome-wide scale [209]. When applied to the peripheral blood, transcriptomics provides a systems approach to on-treatment monitoring with barriers to clinical implementation that could arguably be overcome in the near future, provided the relatively simple platforms of sample acquisition and processing on which this technology rests [210]. The challenge with these high throughput technologies is our ability to process the ever increasing amount of information they yield [211]. The interpretation of dynamic changes in bulk RNA transcripts in a non-closed system such as the peripheral blood, where changes in transcript abundance may in part be driven by timely changes in relative cell frequencies, is even more challenging. Transcriptomics applied to longitudinally collected blood samples could nonetheless be used for the identification of predictive dynamic biomarker signatures of response or of irAE under ICI, the value of which resting in their performance in signaling or predicting outcomes rather than on their interpretability. Changes in blood transcriptomic have been described in several conditions, including autoimmune and infectious diseases, neurological disorders, cardiovascular diseases, but also in both hematological and a wide range of solid malignancies [209]. While the design of the majority of blood transcriptomic studies published to date has been cross-sectional [209], longitudinal studies are increasingly implemented. This is notably illustrated by studies documenting changes in blood transcriptome induced by vaccines [212, 213]. In the context of cancer vaccines, a transcriptomic analysis of peripheral blood mononuclear cells of melanoma patients treated with an adjuvant anti-DEC-NYESO vaccine administered with or without a dendritic cell stimulant fms-like tyrosine kinase 3 ligand (Flt3L), defined the kinetic of immune modulation induced by Flt3L administration (Bhardwaj et al., Nature Cancer, in press). The study described gene signatures associated with the Flt3L induction of an early and sustained immune response, with most dramatic changes occurring at days 8 and 15 of treatment introduction (Bhardwaj et al., Nature Cancer, in press). Changes in blood transcriptome were also described in cancer patients under radiation therapy [214], as well as in melanoma patients under systemic interleukin 2 (IL-2) therapy [215]. Longitudinal blood transcriptomic profiling of patients treated by the tyrosine-kinase inhibitor pazopanib similarly allowed to register a transient but significant systemic immune modulation peaking at the third month of treatment under this *a priori* non-immunologic agent (Rinchai et al., Clinical and Translational Medicine, under revision; preprint in biorxive: https://www.biorxiv.org/content/10.1101/2020.05.01.071613v1). These studies collectively illustrate the potential to register the systemic immunomodulatory effects of a range of different therapeutic agents through the dynamic monitoring of the blood transcriptome. Applying blood transcriptomics to monitor the dynamic systemic immune perturbations associated with anti-CTLA-4 monotherapy and combined anti-CTLA-4 and anti-PD-1 therapy, Rinchai et al. observed a peak of immune modulation at day 8 and 15 with dramatically different transcriptional upregulations of gene modules reflecting TGF-β signaling and TNF-α between treatments (Rinchai et al., SITC 2019, Abstract). The predictive and prognostic value of these ICI treatment induced dynamic changes in blood transcriptome remains to be investigated.

The development of platforms allowing for the comprehensive profiling of additional compartments of a sample, including the proteome, the metabolome and even possibly the microbiome, may, in time, contribute complementary data of potential relevance to patient monitoring under ICI, although the implementation of these technologies in the clinical practice lays most probably further down the road, provided the comparative lag in maturity of the corresponding platforms.

## Conclusions

A better grasp onto the pathways mediating the action of PD-1 and CTLA-4 blockade on the tumor is important to enhance our ability to monitor for their effects in patients in real time, thereby addressing the uncertainty in treatment outcome following their introduction. As illustrated in this review, preclinical studies based on animal models are an important source of progress in this field. These models may however not always accurately reflect human cancer immune biology [216]. The extent to which the reported findings of such studies apply to the clinical setting remains in some instances uncertain, highlighting the importance of translational research based on the immunomonitoring of ICI treated patients in moving forward. In spite of these limitations, the study of the dynamics of the immune system under ICI reveals stark contrast in the way CTLA-4 and PD-1 inhibitors operate their effect on the anti-tumoral immune response. It however appears that both anti-PD-1 and anti-CTLA-4 are capable of inducing a profound remodeling of the tumor microenvironment via their engagement with different immune cell subsets. A comprehensive assessment of the pathways mediating ICI tumor control through systems approaches, allowed for by omics technologies, is likely to be key in the identification of reliable dynamic biomarkers of ICI outcome as well as in patient monitoring under treatment in the near future. Studies based on such technologies have yielded considerable gains in knowledge on mechanisms involved in ICI anti-tumoral action but have also raised in the process new questions stemming from unraveled layers of complexity. Progress along this road is crucial to build rationales for novel therapeutic combinations and to pave the way towards a more personalized approach to immune cancer therapies.

## Data Availability

Not applicable.

## List of Abbreviations

ICIs: Immune checkpoint inhibitors
TME: Tumor microenvironment
CLTA-4: Cytotoxic T-lymphocyte antigen 4
PD-1: Programmed cell death protein 1
PD-L1: Programmed death-ligand 1
TCR: T cell receptor
APC: Antigen presenting cells
TIM-3: T cell immunoglobulin and mucin domain-containing protein 3
GITR: Glucocorticoid-induced tumor necrosis factor receptor
IFN-γ: Interferon gamma
Th1: T helper 1
ICOS: Inducible T cell co-stimulator
HLA: human leukocyte antigen
CCR7: C-C chemokine receptor type 7
PBMC: Peripheral blood mononuclear cells
IRAE: Immune-related adverse event
Tregs: Regulatory T cells
Foxp-3: Forkhead box P3
ADCC: Antibody dependent cell-mediated cytotoxicity
Teffs: Effector T cells
LAG3: Lymphocyte-activation protein 3
TIGIT: T cell immunoreceptor with Ig and ITIM domains.
TNF-α: Tumor necrosis factor alpha.
tcf-1: T cell factor 1.
CXCL13: Chemokine ligand 13.
NSCLC: non-small cell lung carcinoma.
DCB: Durable clinical benefit.
LCMV: chronic lymphocytic choriomeningitis virus
TAB: Tumor associated B cells
Bregs: B regulatory
TGF-β: Transforming growth factor beta
TLS: Tertiary lymphoid structures
MHC: Major histocompatibility complex
MDSCs: Myeloid derived suppressor cells
TAMs: Tumor-associated macrophages
mo-MDSCs: Monocytic MDSCs
PMN-MDSCs: Polymorphonuclear MDSCs
VISTA: V-domain Ig suppressor of T cell activation
FDA: Food and Drug Administration
SNVs: Single nucleotide variants
TMB: Tumor mutational burden
β-2M: Beta-2-microglobulin
PI3K: Phosphoinositide 3-kinase
Flt3L: Fms-like tyrosine kinase 3 ligand
IL-2: Interleukin 2

## References

[1] Larkin J (2019). Five-Year Survival with Combined Nivolumab and Ipilimumab in Advanced Melanoma. N Engl J Med.

[2] Maio M (2015). Five-year survival rates for treatment-naive patients with advanced melanoma who received ipilimumab plus dacarbazine in a phase III trial. J Clin Oncol.

[3] Wolchok JD (2017). Overall Survival with Combined Nivolumab and Ipilimumab in Advanced Melanoma. N Engl J Med.

[4] Robert C (2019). Pembrolizumab versus ipilimumab in advanced melanoma (KEYNOTE-006): post-hoc 5-year results from an open-label, multicentre, randomised, controlled, phase 3 study. Lancet Oncol.

[5] Havel JJ, Chowell D, Chan TA (2019). The evolving landscape of biomarkers for checkpoint inhibitor immunotherapy. Nat Rev Cancer.

[6] Keenan TE, Burke KP, Van Allen EM (2019). Genomic correlates of response to immune checkpoint blockade. Nat Med.

[7] K Choucair et al., TMB: a promising immune-response biomarker, and potential spearhead in advancing targeted therapy trials. *Cancer Gene Ther*, (2020). 10.1038/s41417-020-0174-y 32341410

[8] Chowell D (2018). Patient HLA class I genotype influences cancer response to checkpoint blockade immunotherapy. Science.

[9] Shaikh FY, Gills JJ, Sears CL (2019). Impact of the microbiome on checkpoint inhibitor treatment in patients with non-small cell lung cancer and melanoma. EBioMedicine.

[10] Sivan A (2015). Commensal Bifidobacterium promotes antitumor immunity and facilitates anti-PD-L1 efficacy. Science.

[11] Vetizou M (2015). Anticancer immunotherapy by CTLA-4 blockade relies on the gut microbiota. Science.

[12] Greaves M, Maley CC (2012). Clonal evolution in cancer. Nature.

[13] Yates LR, Campbell PJ (2012). Evolution of the cancer genome. Nat Rev Genet.

[14] Hodgkin PD (2007). A probabilistic view of immunology: drawing parallels with physics. Immunol Cell Biol.

[15] D Duneau et al., Stochastic variation in the initial phase of bacterial infection predicts the probability of survival in D. melanogaster. *Elife* 6, (2017). 10.7554/eLife.28298 PMC5703640 29022878

[16] Lesterhuis WJ (2017). Dynamic versus static biomarkers in cancer immune checkpoint blockade: unravelling complexity. Nat Rev Drug Discov.

[17] Walunas TL (1994). CTLA-4 can function as a negative regulator of T cell activation. Immunity.

[18] Waterhouse P (1995). Lymphoproliferative disorders with early lethality in mice deficient in Ctla-4. Science.

[19] Wei SC (2017). Distinct Cellular Mechanisms Underlie Anti-CTLA-4 and Anti-PD-1 Checkpoint Blockade. Cell.

[20] Gubin MM (2014). Checkpoint blockade cancer immunotherapy targets tumour-specific mutant antigens. Nature.

[21] Fehlings M (2017). Checkpoint blockade immunotherapy reshapes the high-dimensional phenotypic heterogeneity of murine intratumoural neoantigen-specific CD8(+) T cells. Nat Commun.

[22] Ribas A (2009). Dendritic cell vaccination combined with CTLA4 blockade in patients with metastatic melanoma. Clin Cancer Res.

[23] Klein O (2009). Melan-A-specific cytotoxic T cells are associated with tumor regression and autoimmunity following treatment with anti-CTLA-4. Clin Cancer Res.

[24] Chen PL (2016). Analysis of Immune Signatures in Longitudinal Tumor Samples Yields Insight into Biomarkers of Response and Mechanisms of Resistance to Immune Checkpoint Blockade. Cancer Discov.

[25] Ribas A (2009). Intratumoral immune cell infiltrates, FoxP3, and indoleamine 2,3-dioxygenase in patients with melanoma undergoing CTLA4 blockade. Clin Cancer Res.

[26] Ji RR (2012). An immune-active tumor microenvironment favors clinical response to ipilimumab. Cancer Immunol Immunother.

[27] Pedicord VA, Montalvo W, Leiner IM, Allison JP (2011). Single dose of anti-CTLA-4 enhances CD8 + T-cell memory formation, function, and maintenance. Proc Natl Acad Sci U S A.

[28] Felix J (2016). Ipilimumab reshapes T cell memory subsets in melanoma patients with clinical response. Oncoimmunology.

[29] de Coana YP (2017). Ipilimumab treatment decreases monocytic MDSCs and increases CD8 effector memory T cells in long-term survivors with advanced melanoma. Oncotarget.

[30] Huang RR (2011). CTLA4 blockade induces frequent tumor infiltration by activated lymphocytes regardless of clinical responses in humans. Clin Cancer Res.

[31] Restifo NP, Smyth MJ, Snyder A (2016). Acquired resistance to immunotherapy and future challenges. Nat Rev Cancer.

[32] Sharma P, Hu-Lieskovan S, Wargo JA, Ribas A (2017). Primary, Adaptive, and Acquired Resistance to Cancer Immunotherapy. Cell.

[33] Hosoi A (2018). Increased diversity with reduced “diversity evenness” of tumor infiltrating T-cells for the successful cancer immunotherapy. Sci Rep.

[34] Fu T, He Q, Sharma P (2011). The ICOS/ICOSL pathway is required for optimal antitumor responses mediated by anti-CTLA-4 therapy. Cancer Res.

[35] Jiao S (2019). Differences in Tumor Microenvironment Dictate T Helper Lineage Polarization and Response to Immune Checkpoint Therapy. Cell.

[36] von Euw E (2009). CTLA4 blockade increases Th17 cells in patients with metastatic melanoma. J Transl Med.

[37] Yasuda K, Takeuchi Y, Hirota K (2019). The pathogenicity of Th17 cells in autoimmune diseases. Semin Immunopathol.

[38] Chen H (2009). Anti-CTLA-4 therapy results in higher CD4 + ICOShi T cell frequency and IFN-gamma levels in both nonmalignant and malignant prostate tissues. Proc Natl Acad Sci U S A.

[39] Liakou CI (2008). CTLA-4 blockade increases IFNgamma-producing CD4 + ICOShi cells to shift the ratio of effector to regulatory T cells in cancer patients. Proc Natl Acad Sci U S A.

[40] Carthon BC (2010). Preoperative CTLA-4 blockade: tolerability and immune monitoring in the setting of a presurgical clinical trial. Clin Cancer Res.

[41] Ng Tang D (2013). Increased frequency of ICOS + CD4 T cells as a pharmacodynamic biomarker for anti-CTLA-4 therapy. Cancer Immunol Res.

[42] Weber JS (2012). Ipilimumab increases activated T cells and enhances humoral immunity in patients with advanced melanoma. J Immunother.

[43] Kitano S (2013). Enhancement of tumor-reactive cytotoxic CD4 + T cell responses after ipilimumab treatment in four advanced melanoma patients. Cancer Immunol Res.

[44] Vonderheide RH (2010). Tremelimumab in combination with exemestane in patients with advanced breast cancer and treatment-associated modulation of inducible costimulator expression on patient T cells. Clin Cancer Res.

[45] Das R (2015). Combination therapy with anti-CTLA-4 and anti-PD-1 leads to distinct immunologic changes in vivo. J Immunol.

[46] Kavanagh B (2008). CTLA4 blockade expands FoxP3 + regulatory and activated effector CD4 + T cells in a dose-dependent fashion. Blood.

[47] Wang W (2012). Biomarkers on melanoma patient T cells associated with ipilimumab treatment. J Transl Med.

[48] Pico de Coana Y (2013). Ipilimumab treatment results in an early decrease in the frequency of circulating granulocytic myeloid-derived suppressor cells as well as their Arginase1 production. Cancer Immunol Res.

[49] Phan GQ (2003). Cancer regression and autoimmunity induced by cytotoxic T lymphocyte-associated antigen 4 blockade in patients with metastatic melanoma. Proc Natl Acad Sci U S A.

[50] Wistuba-Hamprecht K (2017). Peripheral CD8 effector-memory type 1 T-cells correlate with outcome in ipilimumab-treated stage IV melanoma patients. Eur J Cancer.

[51] Yuan J (2008). CTLA-4 blockade enhances polyfunctional NY-ESO-1 specific T cell responses in metastatic melanoma patients with clinical benefit. Proc Natl Acad Sci U S A.

[52] Hodi FS (2008). Immunologic and clinical effects of antibody blockade of cytotoxic T lymphocyte-associated antigen 4 in previously vaccinated cancer patients. Proc Natl Acad Sci U S A.

[53] Martens A (2016). Increases in Absolute Lymphocytes and Circulating CD4 + and CD8 + T Cells Are Associated with Positive Clinical Outcome of Melanoma Patients Treated with Ipilimumab. Clin Cancer Res.

[54] Kelderman S (2014). Lactate dehydrogenase as a selection criterion for ipilimumab treatment in metastatic melanoma. Cancer Immunol Immunother.

[55] Ku GY (2010). Single-institution experience with ipilimumab in advanced melanoma patients in the compassionate use setting: lymphocyte count after 2 doses correlates with survival. Cancer.

[56] Delyon J (2013). Experience in daily practice with ipilimumab for the treatment of patients with metastatic melanoma: an early increase in lymphocyte and eosinophil counts is associated with improved survival. Ann Oncol.

[57] Sarnaik AA (2011). Extended dose ipilimumab with a peptide vaccine: immune correlates associated with clinical benefit in patients with resected high-risk stage IIIc/IV melanoma. Clin Cancer Res.

[58] Ribas A (2010). Imaging of CTLA4 blockade-induced cell replication with (18)F-FLT PET in patients with advanced melanoma treated with tremelimumab. J Nucl Med.

[59] Robert L (2014). CTLA4 blockade broadens the peripheral T-cell receptor repertoire. Clin Cancer Res.

[60] Cha E (2014). Improved survival with T cell clonotype stability after anti-CTLA-4 treatment in cancer patients. Sci Transl Med.

[61] Oh DY (2017). Immune Toxicities Elicted by CTLA-4 Blockade in Cancer Patients Are Associated with Early Diversification of the T-cell Repertoire. Cancer Res.

[62] Arakawa A (2019). Clonality of CD4(+) Blood T Cells Predicts Longer Survival With CTLA4 or PD-1 Checkpoint Inhibition in Advanced Melanoma. Front Immunol.

[63] Messaoudi I, Guevara Patino JA, Dyall R, LeMaoult J. J. Nikolich-Zugich, Direct link between mhc polymorphism, T cell avidity, and diversity in immune defense. Science. 2002;298:1797–800. 10.1126/science.1076064 12459592

[64] Kvistborg P (2014). Anti-CTLA-4 therapy broadens the melanoma-reactive CD8 + T cell response. Sci Transl Med.

[65] Subudhi SK (2016). Clonal expansion of CD8 T cells in the systemic circulation precedes development of ipilimumab-induced toxicities. Proc Natl Acad Sci U S A.

[66] Postow MA (2015). Peripheral T cell receptor diversity is associated with clinical outcomes following ipilimumab treatment in metastatic melanoma. J Immunother Cancer.

[67] Page DB (2016). Deep Sequencing of T-cell Receptor DNA as a Biomarker of Clonally Expanded TILs in Breast Cancer after Immunotherapy. Cancer Immunol Res.

[68] Mougiakakos D, Choudhury A, Lladser A, Kiessling R, Johansson CC (2010). Regulatory T cells in cancer. Adv Cancer Res.

[69] Wei T, Zhong W, Li Q (2020). Role of heterogeneous regulatory T cells in the tumor microenvironment. Pharmacol Res.

[70] Facciabene A, Motz GT, Coukos G (2012). T-regulatory cells: key players in tumor immune escape and angiogenesis. Cancer Res.

[71] Takahashi T (2000). Immunologic self-tolerance maintained by CD25(+)CD4(+) regulatory T cells constitutively expressing cytotoxic T lymphocyte-associated antigen 4. J Exp Med.

[72] Zheng Y, Rudensky AY (2007). Foxp3 in control of the regulatory T cell lineage. Nat Immunol.

[73] Chen C, Rowell EA, Thomas RM, Hancock WW, Wells AD (2006). Transcriptional regulation by Foxp3 is associated with direct promoter occupancy and modulation of histone acetylation. J Biol Chem.

[74] Read S (2006). Blockade of CTLA-4 on CD4 + CD25 + regulatory T cells abrogates their function in vivo. J Immunol.

[75] Friedline RH (2009). CD4 + regulatory T cells require CTLA-4 for the maintenance of systemic tolerance. J Exp Med.

[76] Paterson AM (2015). Deletion of CTLA-4 on regulatory T cells during adulthood leads to resistance to autoimmunity. J Exp Med.

[77] Peggs KS, Quezada SA, Chambers CA, Korman AJ, Allison JP (2009). Blockade of CTLA-4 on both effector and regulatory T cell compartments contributes to the antitumor activity of anti-CTLA-4 antibodies. J Exp Med.

[78] Selby MJ (2013). Anti-CTLA-4 antibodies of IgG2a isotype enhance antitumor activity through reduction of intratumoral regulatory T cells. Cancer Immunol Res.

[79] Tang F, Du X, Liu M, Zheng P, Liu Y (2018). Anti-CTLA-4 antibodies in cancer immunotherapy: selective depletion of intratumoral regulatory T cells or checkpoint blockade?. Cell Biosci.

[80] Simpson TR (2013). Fc-dependent depletion of tumor-infiltrating regulatory T cells co-defines the efficacy of anti-CTLA-4 therapy against melanoma. J Exp Med.

[81] Arce Vargas F (2018). Fc Effector Function Contributes to the Activity of Human Anti-CTLA-4 Antibodies. Cancer Cell.

[82] Liu Y, Zheng P (2018). How Does an Anti-CTLA-4 Antibody Promote Cancer Immunity?. Trends Immunol.

[83] Pages F (2018). International validation of the consensus Immunoscore for the classification of colon cancer: a prognostic and accuracy study. Lancet.

[84] Saito T (2016). Two FOXP3(+)CD4(+) T cell subpopulations distinctly control the prognosis of colorectal cancers. Nat Med.

[85] Quezada SA, Peggs KS, Curran MA, Allison JP (2006). CTLA4 blockade and GM-CSF combination immunotherapy alters the intratumor balance of effector and regulatory T cells. J Clin Invest.

[86] Du X (2018). A reappraisal of CTLA-4 checkpoint blockade in cancer immunotherapy. Cell Res.

[87] Bjoern J (2016). Immunological correlates of treatment and response in stage IV malignant melanoma patients treated with Ipilimumab. Oncoimmunology.

[88] Tarhini AA (2014). Immune monitoring of the circulation and the tumor microenvironment in patients with regionally advanced melanoma receiving neoadjuvant ipilimumab. PLoS One.

[89] Khan S (2011). Tremelimumab (anti-CTLA4) mediates immune responses mainly by direct activation of T effector cells rather than by affecting T regulatory cells. Clin Immunol.

[90] Simeone E (2014). Immunological and biological changes during ipilimumab treatment and their potential correlation with clinical response and survival in patients with advanced melanoma. Cancer Immunol Immunother.

[91] Maker AV, Attia P, Rosenberg SA (2005). Analysis of the cellular mechanism of antitumor responses and autoimmunity in patients treated with CTLA-4 blockade. J Immunol.

[92] Romano E (2015). Ipilimumab-dependent cell-mediated cytotoxicity of regulatory T cells ex vivo by nonclassical monocytes in melanoma patients. Proc Natl Acad Sci U S A.

[93] Sharma A (2019). Anti-CTLA-4 Immunotherapy Does Not Deplete FOXP3(+) Regulatory T Cells (Tregs) in Human Cancers-Response. Clin Cancer Res.

[94] Quezada SA, Peggs KS (2019). Lost in Translation: Deciphering the Mechanism of Action of Anti-human CTLA-4. Clin Cancer Res.

[95] Herbst RS (2014). Predictive correlates of response to the anti-PD-L1 antibody MPDL3280A in cancer patients. Nature.

[96] Tumeh PC (2014). PD-1 blockade induces responses by inhibiting adaptive immune resistance. Nature.

[97] Ribas A (2016). PD-1 Blockade Expands Intratumoral Memory T Cells. Cancer Immunol Res.

[98] Verma V (2019). PD-1 blockade in subprimed CD8 cells induces dysfunctional PD-1(+)CD38(hi) cells and anti-PD-1 resistance. Nat Immunol.

[99] CS Grasso et al., Conserved Interferon-gamma Signaling Drives Clinical Response to Immune Checkpoint Blockade Therapy in Melanoma. *Cancer Cell*, (2020). 10.1016/j.ccell.2020.08.005 PMC7872287 32916126

[100] Huang AC (2017). T-cell invigoration to tumour burden ratio associated with anti-PD-1 response. Nature.

[101] Kamphorst AO (2017). Proliferation of PD-1 + CD8 T cells in peripheral blood after PD-1-targeted therapy in lung cancer patients. Proc Natl Acad Sci U S A.

[102] Kim KH (2019). The First-week Proliferative Response of Peripheral Blood PD-1(+)CD8(+) T Cells Predicts the Response to Anti-PD-1 Therapy in Solid Tumors. Clin Cancer Res.

[103] Kamada T (2019). PD-1(+) regulatory T cells amplified by PD-1 blockade promote hyperprogression of cancer. Proc Natl Acad Sci U S A.

[104] Woods DM (2018). Decreased Suppression and Increased Phosphorylated STAT3 in Regulatory T Cells are Associated with Benefit from Adjuvant PD-1 Blockade in Resected Metastatic Melanoma. Clin Cancer Res.

[105] Zappasodi R (2018). Non-conventional Inhibitory CD4(+)Foxp3(-)PD-1(hi) T Cells as a Biomarker of Immune Checkpoint Blockade Activity. Cancer Cell.

[106] Riaz N (2017). Tumor and Microenvironment Evolution during Immunotherapy with Nivolumab. Cell.

[107] J Han et al. TCR Repertoire Diversity of Peripheral PD-1(+)CD8(+) T Cells Predicts Clinical Outcomes after Immunotherapy in Patients with Non-Small Cell Lung Cancer. Cancer Immunol Res. 2020;8:146–54. 10.1158/2326-6066.CIR-19-0398 31719056

[108] Snyder A (2017). Contribution of systemic and somatic factors to clinical response and resistance to PD-L1 blockade in urothelial cancer: An exploratory multi-omic analysis. PLoS Med.

[109] Gebhardt C (2015). Myeloid Cells and Related Chronic Inflammatory Factors as Novel Predictive Markers in Melanoma Treatment with Ipilimumab. Clin Cancer Res.

[110] Das R (2018). Early B cell changes predict autoimmunity following combination immune checkpoint blockade. J Clin Invest.

[111] Topalian SL, Drake CG, Pardoll DM (2012). Targeting the PD-1/B7-H1(PD-L1) pathway to activate anti-tumor immunity. Curr Opin Immunol.

[112] Latchman Y (2001). PD-L2 is a second ligand for PD-1 and inhibits T cell activation. Nat Immunol.

[113] Paley MA (2012). Progenitor and terminal subsets of CD8 + T cells cooperate to contain chronic viral infection. Science.

[114] Blackburn SD (2009). Coregulation of CD8 + T cell exhaustion by multiple inhibitory receptors during chronic viral infection. Nat Immunol.

[115] Thommen DS (2018). A transcriptionally and functionally distinct PD-1(+) CD8(+) T cell pool with predictive potential in non-small-cell lung cancer treated with PD-1 blockade. Nat Med.

[116] Li H (2019). Dysfunctional CD8 T Cells Form a Proliferative, Dynamically Regulated Compartment within Human Melanoma. Cell.

[117] Pauken KE (2016). Epigenetic stability of exhausted T cells limits durability of reinvigoration by PD-1 blockade. Science.

[118] Philip M (2017). Chromatin states define tumour-specific T cell dysfunction and reprogramming. Nature.

[119] Ghoneim HE (2017). De Novo Epigenetic Programs Inhibit PD-1 Blockade-Mediated T Cell Rejuvenation. Cell.

[120] Im SJ (2016). Defining CD8 + T cells that provide the proliferative burst after PD-1 therapy. Nature.

[121] Jadhav RR (2019). Epigenetic signature of PD-1 + TCF1 + CD8 T cells that act as resource cells during chronic viral infection and respond to PD-1 blockade. Proc Natl Acad Sci U S A.

[122] I Siddiqui et al., Intratumoral Tcf1(+)PD-1(+)CD8(+) T Cells with Stem-like Properties Promote Tumor Control in Response to Vaccination and Checkpoint Blockade Immunotherapy. Immunity 50, 195–211 e110 (2019). 10.1016/j.immuni.2018.12.021 30635237

[123] Miller BC (2019). Subsets of exhausted CD8(+) T cells differentially mediate tumor control and respond to checkpoint blockade. Nat Immunol.

[124] Yost KE (2019). Clonal replacement of tumor-specific T cells following PD-1 blockade. Nat Med.

[125] Huang AC (2019). A single dose of neoadjuvant PD-1 blockade predicts clinical outcomes in resectable melanoma. Nat Med.

[126] Sade-Feldman M (2018). Defining T Cell States Associated with Response to Checkpoint Immunotherapy in Melanoma. Cell.

[127] Kurtulus S (2019). Checkpoint Blockade Immunotherapy Induces Dynamic Changes in PD-1(-)CD8(+) Tumor-Infiltrating T Cells. Immunity.

[128] Ganesan AP (2017). Tissue-resident memory features are linked to the magnitude of cytotoxic T cell responses in human lung cancer. Nat Immunol.

[129] Spitzer MH (2017). Systemic Immunity Is Required for Effective Cancer Immunotherapy. Cell.

[130] Forde PM, Chaft JE, Pardoll DM (2018). Neoadjuvant PD-1 Blockade in Resectable Lung Cancer. N Engl J Med.

[131] M Merhi et al., Persistent anti-NY-ESO-1-specific T cells and expression of differential biomarkers in a patient with metastatic gastric cancer benefiting from combined radioimmunotherapy treatment: a case report. *J Immunother Cancer* 8, (2020). 10.1136/jitc-2020-001278 PMC7484873 32913031

[132] Sakaguchi S, Miyara M, Costantino CM, Hafler DA (2010). FOXP3 + regulatory T cells in the human immune system. Nat Rev Immunol.

[133] DE Lowther et al., PD-1 marks dysfunctional regulatory T cells in malignant gliomas. *JCI Insight* 1, (2016). 10.1172/jci.insight.85935 PMC4864991 27182555

[134] Togashi Y, Shitara K, Nishikawa H (2019). Regulatory T cells in cancer immunosuppression - implications for anticancer therapy. Nat Rev Clin Oncol.

[135] Duraiswamy J, Kaluza KM, Freeman GJ, Coukos G (2013). Dual blockade of PD-1 and CTLA-4 combined with tumor vaccine effectively restores T-cell rejection function in tumors. Cancer Res.

[136] Zhou Q (2010). Program death-1 signaling and regulatory T cells collaborate to resist the function of adoptively transferred cytotoxic T lymphocytes in advanced acute myeloid leukemia. Blood.

[137] Park HJ (2015). PD-1 upregulated on regulatory T cells during chronic virus infection enhances the suppression of CD8 + T cell immune response via the interaction with PD-L1 expressed on CD8 + T cells. J Immunol.

[138] Kitazawa Y (2007). Involvement of the programmed death-1/programmed death-1 ligand pathway in CD4 + CD25 + regulatory T-cell activity to suppress alloimmune responses. Transplantation.

[139] Yoshida K (2020). Anti-PD-1 antibody decreases tumour-infiltrating regulatory T cells. BMC Cancer.

[140] Wang W (2009). PD1 blockade reverses the suppression of melanoma antigen-specific CTL by CD4 + CD25(Hi) regulatory T cells. Int Immunol.

[141] Amarnath S (2011). The PDL1-PD1 axis converts human TH1 cells into regulatory T cells. Sci Transl Med.

[142] Francisco LM (2009). PD-L1 regulates the development, maintenance, and function of induced regulatory T cells. J Exp Med.

[143] Stathopoulou C (2018). PD-1 Inhibitory Receptor Downregulates Asparaginyl Endopeptidase and Maintains Foxp3 Transcription Factor Stability in Induced Regulatory T Cells. Immunity.

[144] Wong M, La Cava A, Hahn BH (2013). Blockade of programmed death-1 in young (New Zealand Black x New Zealand White)F1 mice promotes the suppressive capacity of CD4 + regulatory T cells protecting from lupus-like disease. J Immunol.

[145] Dodagatta-Marri E (2019). alpha-PD-1 therapy elevates Treg/Th balance and increases tumor cell pSmad3 that are both targeted by alpha-TGFbeta antibody to promote durable rejection and immunity in squamous cell carcinomas. J Immunother Cancer.

[146] Franceschini D (2009). PD-L1 negatively regulates CD4 + CD25 + Foxp3 + Tregs by limiting STAT-5 phosphorylation in patients chronically infected with HCV. J Clin Invest.

[147] Penaloza-MacMaster P, Provine NM, Blass E, Barouch DH (2015). CD4 T Cell Depletion Substantially Augments the Rescue Potential of PD-L1 Blockade for Deeply Exhausted CD8 T Cells. J Immunol.

[148] Xiong Y (2020). Immunological effects of nivolumab immunotherapy in patients with oral cavity squamous cell carcinoma. BMC Cancer.

[149] Akhurst RJ, Hata A (2012). Targeting the TGFbeta signalling pathway in disease. Nat Rev Drug Discov.

[150] Cabrita R (2020). Tertiary lymphoid structures improve immunotherapy and survival in melanoma. Nature.

[151] Erdag G (2012). Immunotype and immunohistologic characteristics of tumor-infiltrating immune cells are associated with clinical outcome in metastatic melanoma. Cancer Res.

[152] Griss J (2019). B cells sustain inflammation and predict response to immune checkpoint blockade in human melanoma. Nat Commun.

[153] Rodriguez-Pinto D (2005). B cells as antigen presenting cells. Cell Immunol.

[154] Nielsen JS (2012). CD20 + tumor-infiltrating lymphocytes have an atypical CD27- memory phenotype and together with CD8 + T cells promote favorable prognosis in ovarian cancer. Clin Cancer Res.

[155] Mauri C, Menon M (2017). Human regulatory B cells in health and disease: therapeutic potential. J Clin Invest.

[156] Wang X (2019). PD-1-expressing B cells suppress CD4(+) and CD8(+) T cells via PD-1/PD-L1-dependent pathway. Mol Immunol.

[157] Wouters MCA, Nelson BH (2018). Prognostic Significance of Tumor-Infiltrating B Cells and Plasma Cells in Human Cancer. Clin Cancer Res.

[158] Neyt K, Perros F, GeurtsvanKessel CH, Hammad H, Lambrecht BN (2012). Tertiary lymphoid organs in infection and autoimmunity. Trends Immunol.

[159] Baddoura FK (2005). Lymphoid neogenesis in murine cardiac allografts undergoing chronic rejection. Am J Transplant.

[160] Helmink BA (2020). B cells and tertiary lymphoid structures promote immunotherapy response. Nature.

[161] Petitprez F (2020). B cells are associated with survival and immunotherapy response in sarcoma. Nature.

[162] DP Hollern et al., B Cells and T Follicular Helper Cells Mediate Response to Checkpoint Inhibitors in High Mutation Burden Mouse Models of Breast Cancer. Cell 179, 1191–206 e1121 (2019). 10.1016/j.cell.2019.10.028 PMC6911685 31730857

[163] Bindea G (2013). Spatiotemporal dynamics of intratumoral immune cells reveal the immune landscape in human cancer. Immunity.

[164] Gu-Trantien C (2013). CD4(+) follicular helper T cell infiltration predicts breast cancer survival. J Clin Invest.

[165] Amaria RN (2018). Neoadjuvant immune checkpoint blockade in high-risk resectable melanoma. Nat Med.

[166] Nakamura K, Smyth MJ (2020). Myeloid immunosuppression and immune checkpoints in the tumor microenvironment. Cell Mol Immunol.

[167] Martinez FO, Gordon S (2014). The M1 and M2 paradigm of macrophage activation: time for reassessment. F1000Prime Rep.

[168] Gubin MM (2018). High-Dimensional Analysis Delineates Myeloid and Lymphoid Compartment Remodeling during Successful Immune-Checkpoint Cancer Therapy. Cell.

[169] Xiong H (2019). Anti-PD-L1 Treatment Results in Functional Remodeling of the Macrophage Compartment. Cancer Res.

[170] Zhu Y (2014). CSF1/CSF1R blockade reprograms tumor-infiltrating macrophages and improves response to T-cell checkpoint immunotherapy in pancreatic cancer models. Cancer Res.

[171] Beavis PA (2018). Dual PD-1 and CTLA-4 Checkpoint Blockade Promotes Antitumor Immune Responses through CD4(+)Foxp3(-) Cell-Mediated Modulation of CD103(+) Dendritic Cells. Cancer Immunol Res.

[172] Laurent S (2010). CTLA-4 is expressed by human monocyte-derived dendritic cells and regulates their functions. Hum Immunol.

[173] Liu Y (2009). Regulation of arginase I activity and expression by both PD-1 and CTLA-4 on the myeloid-derived suppressor cells. Cancer Immunol Immunother.

[174] Rodriguez PC (2004). Arginase I production in the tumor microenvironment by mature myeloid cells inhibits T-cell receptor expression and antigen-specific T-cell responses. Cancer Res.

[175] Strauss L, et al., Targeted deletion of PD-1 in myeloid cells induces antitumor immunity. *Sci Immunol* 5, (2020). 10.1126/sciimmunol.aay1863 PMC7183328 31901074

[176] Kim SH (2017). Phenformin Inhibits Myeloid-Derived Suppressor Cells and Enhances the Anti-Tumor Activity of PD-1 Blockade in Melanoma. J Invest Dermatol.

[177] Kim K (2014). Eradication of metastatic mouse cancers resistant to immune checkpoint blockade by suppression of myeloid-derived cells. Proc Natl Acad Sci U S A.

[178] Orillion A (2017). Entinostat Neutralizes Myeloid-Derived Suppressor Cells and Enhances the Antitumor Effect of PD-1 Inhibition in Murine Models of Lung and Renal Cell Carcinoma. Clin Cancer Res.

[179] Clavijo PE (2017). Resistance to CTLA-4 checkpoint inhibition reversed through selective elimination of granulocytic myeloid cells. Oncotarget.

[180] Davis RJ (2017). Anti-PD-L1 Efficacy Can Be Enhanced by Inhibition of Myeloid-Derived Suppressor Cells with a Selective Inhibitor of PI3Kdelta/gamma. Cancer Res.

[181] Meyer C (2014). Frequencies of circulating MDSC correlate with clinical outcome of melanoma patients treated with ipilimumab. Cancer Immunol Immunother.

[182] Gabrilovich DI, Nagaraj S (2009). Myeloid-derived suppressor cells as regulators of the immune system. Nat Rev Immunol.

[183] Krieg C (2018). High-dimensional single-cell analysis predicts response to anti-PD-1 immunotherapy. Nat Med.

[184] Bonomi M (2019). Circulating immune biomarkers as predictors of the response to pembrolizumab and weekly low dose carboplatin and paclitaxel in NSCLC and poor PS: An interim analysis. Oncol Lett.

[185] Biswas SK, Mantovani A (2010). Macrophage plasticity and interaction with lymphocyte subsets: cancer as a paradigm. Nat Immunol.

[186] Koyama S (2016). Adaptive resistance to therapeutic PD-1 blockade is associated with upregulation of alternative immune checkpoints. Nat Commun.

[187] Curran MA, Montalvo W, Yagita H, Allison JP (2010). PD-1 and CTLA-4 combination blockade expands infiltrating T cells and reduces regulatory T and myeloid cells within B16 melanoma tumors. Proc Natl Acad Sci U S A.

[188] Gao J (2017). VISTA is an inhibitory immune checkpoint that is increased after ipilimumab therapy in patients with prostate cancer. Nat Med.

[189] Reese Z, Straubhar A, Pal SK, Agarwal N (2015). Ipilimumab in the treatment of prostate cancer. Future Oncol.

[190] Liu J (2015). Immune-checkpoint proteins VISTA and PD-1 nonredundantly regulate murine T-cell responses. Proc Natl Acad Sci U S A.

[191] Motzer RJ (2018). Nivolumab plus Ipilimumab versus Sunitinib in Advanced Renal-Cell Carcinoma. N Engl J Med.

[192] Hellmann MD (2019). Nivolumab plus Ipilimumab in Advanced Non-Small-Cell Lung Cancer. N Engl J Med.

[193] Spranger S (2014). Mechanism of tumor rejection with doublets of CTLA-4, PD-1/PD-L1, or IDO blockade involves restored IL-2 production and proliferation of CD8(+) T cells directly within the tumor microenvironment. J Immunother Cancer.

[194] Wei SC (2019). Negative Co-stimulation Constrains T Cell Differentiation by Imposing Boundaries on Possible Cell States. Immunity.

[195] Weber JS (2016). Sequential administration of nivolumab and ipilimumab with a planned switch in patients with advanced melanoma (CheckMate 064): an open-label, randomised, phase 2 trial. Lancet Oncol.

[196] Schreiber RD, Old LJ, Smyth MJ (2011). Cancer immunoediting: integrating immunity’s roles in cancer suppression and promotion. Science.

[197] O’Donnell JS, Teng MWL, Smyth MJ (2019). Cancer immunoediting and resistance to T cell-based immunotherapy. Nat Rev Clin Oncol.

[198] Dunn GP, Old LJ, Schreiber RD (2004). The immunobiology of cancer immunosurveillance and immunoediting. Immunity.

[199] Rooney MS, Shukla SA, Wu CJ, Getz G, Hacohen N (2015). Molecular and genetic properties of tumors associated with local immune cytolytic activity. Cell.

[200] Sade-Feldman M (2017). Resistance to checkpoint blockade therapy through inactivation of antigen presentation. Nat Commun.

[201] Zaretsky JM (2016). Mutations Associated with Acquired Resistance to PD-1 Blockade in Melanoma. N Engl J Med.

[202] Spranger S, Gajewski TF (2018). Impact of oncogenic pathways on evasion of antitumour immune responses. Nat Rev Cancer.

[203] Nsengimana J (2018). beta-Catenin-mediated immune evasion pathway frequently operates in primary cutaneous melanomas. J Clin Invest.

[204] Grasso CS (2018). Genetic Mechanisms of Immune Evasion in Colorectal Cancer. Cancer Discov.

[205] Gao J, et al, Loss of IFN-gamma Pathway Genes in Tumor Cells as a Mechanism of Resistance to Anti-CTLA-4 Therapy. *Cell*167, 397–404 e399 (2016). 10.1016/j.cell.2016.08.069 PMC5088716 27667683

[206] Jimenez-Sanchez A (2017). Heterogeneous Tumor-Immune Microenvironments among Differentially Growing Metastases in an Ovarian Cancer Patient. Cell.

[207] Angelova M (2018). Evolution of Metastases in Space and Time under Immune Selection. Cell.

[208] Westerhoff HV, Palsson BO (2004). The evolution of molecular biology into systems biology. Nat Biotechnol.

[209] Chaussabel D (2015). Assessment of immune status using blood transcriptomics and potential implications for global health. Semin Immunol.

[210] Chaussabel D, Baldwin N (2014). Democratizing systems immunology with modular transcriptional repertoire analyses. Nat Rev Immunol.

[211] Chaussabel D, Pulendran B (2015). A vision and a prescription for big data-enabled medicine. Nat Immunol.

[212] Nakaya HI, Pulendran B. Vaccinology in the era of high-throughput biology. *Philos Trans R Soc Lond B Biol Sci* 370, (2015). 10.1098/rstb.2014.0146 PMC4527391 25964458

[213] Obermoser G (2013). Systems scale interactive exploration reveals quantitative and qualitative differences in response to influenza and pneumococcal vaccines. Immunity.

[214] Abend M (2016). Examining Radiation-Induced In Vivo and In Vitro Gene Expression Changes of the Peripheral Blood in Different Laboratories for Biodosimetry Purposes: First RENEB Gene Expression Study. Radiat Res.

[215] Panelli MC (2002). Gene-expression profiling of the response of peripheral blood mononuclear cells and melanoma metastases to systemic IL-2 administration. Genome Biol.

[216] Hegde PS, Chen DS (2020). Top 10 Challenges in Cancer Immunotherapy. Immunity.

